# The Dietary Polyphenol Epigallocatechin Gallate Delays Biological Aging by Activating Peroxisome Biogenesis and Peroxisomal Fatty Acid β‐Oxidation

**DOI:** 10.1111/acel.70795

**Published:** 2026-11-02

**Authors:** Xiaoming Huang, Yanfang Yang, Feng Huang, Wenwen Lin, Qiaowei Li, Yin Yuan, Pengli Zhu

**Affiliations:** ^1^ Shengli Clinical Medical College of Fujian Medical University Fuzhou China; ^2^ Fuzhou University Affiliated Provincial Hospital Fuzhou China; ^3^ Fujian Key Laboratory of Geriatrics Fuzhou China; ^4^ Fujian Provincial Institute of Clinical Geriatrics Fuzhou China

**Keywords:** aging, *Caenorhabditis elegans*, epigallocatechin gallate, lipid metabolism, longevity, nutrition surveys, peroxisomes

## Abstract

Epigallocatechin gallate (EGCG), a major tea polyphenol, has been proposed to promote healthy aging, but population‐level evidence and underlying mechanisms remain unclear. We analyzed 10,977 participants from the National Health and Nutrition Examination Survey (NHANES) and conducted mechanistic studies in *Caenorhabditis elegans* and AML12 hepatocytes. Compared with non‐consumers, both lower and higher EGCG intake categories were associated with lower PhenoAge acceleration, lower all‐cause mortality, and higher estimated residual life expectancy. In *C. elegans* , EGCG supplementation extended lifespan and health span. Transcriptomics revealed upregulation of peroxisome‐related genes. EGCG enhanced peroxisome biogenesis, as shown by increased peroxisome abundance, elevated *prx‐5* and *prx‐19* mRNA levels, and increases in PRX‐5 and PRX‐19 proteins. This increase in peroxisome capacity was accompanied by enhanced peroxisomal fatty acid β‐oxidation, with increased mRNA levels of *acox‐1.2*, *acox‐3*, and *dhs‐28*, increased ACOX‐3 protein, and reduced very long‐chain fatty acids (VLCFAs). Lipidomics showed enhanced processing of VLCFAs and accumulation of shorter‐chain intermediates. Knockdown of *prx‐5*, *acox‐1.2*, or *dhs‐28* abolished EGCG‐induced lifespan and health span benefits. In doxorubicin‐induced senescent AML12 hepatocytes, EGCG reduced the proportion of SA‐β‐gal‐positive cells and decreased p21 and p16 protein levels, while increasing peroxisome abundance and peroxisome‐related proteins, including the biogenesis factors PEX5 and PEX19, the membrane transporters ABCD1 and PMP70, and the β‐oxidation enzymes ACOX1, HSD17B4, EHHADH, and ACAA1. Conversely, the anti‐senescent effects of EGCG were attenuated by *Pex5* or *Acox1* knockdown. Collectively, dietary EGCG intake is associated with slower biological aging, while experimental findings support peroxisome biogenesis and peroxisomal fatty acid β‐oxidation in EGCG‐mediated anti‐aging effects.

## Graphical abstract

Dietary EGCG intake was associated with slower biological aging, lower mortality risk, and longer residual life expectancy in humans. Mechanistic studies in *C. elegans* and AML12 hepatocytes support peroxisome biogenesis and peroxisomal fatty acid β‐oxidation as key contributors to EGCG‐related longevity and anti‐senescent effects.

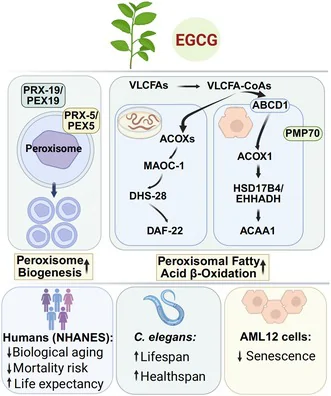

## 1 Introduction

Aging is characterized by a progressive decline in tissue integrity and physiological function and is the dominant risk factor for major chronic conditions (Cai et al. 2022). Identifying safe, scalable strategies to slow functional decline and extend health span, defined as the period of good health (Campos et al. 2023), therefore remains an important goal.

Dietary flavonoids have shown geroprotective effects in experimental models, and population studies associate higher intake with lower risk of all‐cause mortality or slower biological aging (Chen, Wang, and Wei 2024; Zhong et al. 2024; Zhou et al. 2023). Among them, catechins have been widely studied, with epigallocatechin gallate (EGCG) as the most abundant catechin in green tea (Zhang et al. 2024). EGCG has been reported to extend lifespan and attenuate age‐related phenotypes via enhanced mitochondrial function, reduced oxidative stress, and suppressed chronic inflammation (Chen, Wang, and Wei 2024; Wang et al. 2023; Yuan et al. 2020). However, the effects of EGCG on aging appear context‐dependent, and the mechanisms underlying its organism‐level geroprotective benefits remain incompletely understood. Furthermore, evidence linking dietary EGCG exposure to quantitative human biological aging measures is limited, making its public health relevance less clear.

Peroxisomes are essential single‐membrane‐bound organelles and major sites for the β‐oxidation of very long‐chain fatty acids (VLCFAs) and reactive oxygen species (ROS) metabolism (Wanders et al. 2023). Peroxisome biogenesis is a conserved and dynamic process enabling cells to adjust the abundance and metabolic capacity of these organelles in response to physiological demands. Peroxisomal VLCFA β‐oxidation progressively shortens fatty acyl chains through sequential reactions catalyzed by acyl‐CoA oxidases (ACOXs), multifunctional enzymes, and thiolases, ultimately generating chain‐shortened acyl‐CoAs and acetyl‐CoA. ACOXs generate hydrogen peroxide (H_2_O_2_) as a byproduct, while catalase contributes to its removal and redox homeostasis (Wanders et al. 2023). Aging is associated with impaired peroxisome biogenesis and reduced VLCFA β‐oxidation, reflecting broader peroxisomal dysfunction that may contribute to age‐related redox dysregulation (Guo et al. 2025; Kim and Bai 2022; Vercaemst et al. 2026), whereas enhancing peroxisome biogenesis and β‐oxidation can ameliorate aging phenotypes and extend lifespan (Guo et al. 2025; Huang et al. 2020; Wu et al. 2023). EGCG has been reported to increase ACOX expression in canine hepatocytes and *acox‐1.3* expression in *Caenorhabditis elegans* (Ding et al. 2020; Zhang et al. 2023). Nonetheless, whether EGCG exerts geroprotective effects by upregulating peroxisome biogenesis and β‐oxidation remains unexplored.

To assess the association of dietary EGCG with human aging and its geroprotective effects, we integrated human epidemiology with mechanistic studies in *C. elegans* and mammalian cells. Using National Health and Nutrition Examination Survey (NHANES) data, we evaluated the association between dietary EGCG intake and phenotypic age (PhenoAge) acceleration, a robust biomarker of biological aging (Liu et al. 2018). In *C. elegans* , a well‐established aging model (Kirchweger et al. 2023), we tested whether EGCG extends lifespan and health span and whether these effects require peroxisome biogenesis and peroxisomal β‐oxidation. We further examined whether these potential mechanisms extend to mammalian cells using AML12 mouse hepatocytes, given that the liver is rich in peroxisomes involved in VLCFA degradation (Wanders et al. 2023). Together, this multi‐modal approach shows that dietary EGCG is associated with lower biological aging in humans and may promote longevity and delay cellular senescence through peroxisome‐centered mechanisms.

## 2 Results

### 2.1 Baseline Characteristics and Catechin Selection

The 10,977 participants (48.1% male, median age 47 years) had a median PhenoAge of 43 years (interquartile range [IQR]: 29–57), and a median PhenoAge acceleration of −1.5 years (IQR: −5.3 to 2.6; Table 1). The individual catechins exhibited moderate‐to‐strong intercorrelations (Spearman correlation coefficients = 0.57–0.90; *p* < 0.001; Figure 1A), likely due to shared dietary sources. To identify the catechin with the greatest relative predictive contribution to PhenoAge acceleration, we applied complementary feature‐ranking approaches, including partial least squares (PLS) regression and random forest modeling. Both analyses ranked EGCG as the top‐ranked catechin, with the highest variable importance in projection (VIP = 1.897) in the PLS regression and the greatest percent increase in mean squared error (%IncMSE = 54.62%) in random forest modeling (Figure 1B,C). Consequently, EGCG was selected for subsequent analyses.

**TABLE 1:** Baseline characteristics of participants by categories of EGCG intake (*N* = 10,977).

Variables	Overall (*N* = 10,977)	Categories of EGCG intake (mg/day)			*p*
		Zero (0), *N* = 3854	Low (≤ 1.42), *N* = 3562	High (> 1.42), *N* = 3561	
General characteristics					
Age (years)	47 (33, 60)	43 (30, 57)	49 (35, 62)	49 (36, 61)	**< 0.001**
Male (%)	5368 (48.1%)	2078 (53.8%)	1679 (46.2%)	1611 (44.3%)	**< 0.001**
Race, Non‐Hispanic White (%)	5371 (69.9%)	1839 (67.4%)	1651 (69.3%)	1881 (72.7%)	**0.011**
PIR (%)					**< 0.001**
< 1.3	3220 (20.1%)	1381 (25.9%)	952 (17.2%)	887 (17.1%)	
1.3–3.5	4316 (34.9%)	1534 (37.2%)	1448 (33.7%)	1334 (33.8%)	
> 3.5	3441 (45.0%)	939 (36.9%)	1162 (49.1%)	1340 (49.1%)	
PhenoAge acceleration					
PhenoAge acceleration (years)	−1.5 (−5.3, 2.6)	−0.8 (−4.4, 3.6)	−1.9 (−5.7, 2.2)	−1.8 (−5.8, 2.0)	**< 0.001**
Accelerated aging (%)	4559 (39.4%)	1796 (44.4%)	1406 (37.9%)	1357 (35.9%)	**< 0.001**
Lifestyle factors					
Smoking (%)	8727 (80.6%)	2728 (70.6%)	3064 (87.8%)	2935 (83.4%)	**< 0.001**
Alcohol consumption (%)	7211 (69.9%)	2634 (72.8%)	2332 (70.1%)	2245 (66.9%)	**0.012**
Physical activity (%)					**0.012**
Low	4332 (33.8%)	1584 (35.5%)	1373 (32.6%)	1375 (33.4%)	
Moderate	3153 (30.9%)	982 (27.2%)	1061 (31.9%)	1110 (33.4%)	
Vigorous	3492 (35.3%)	1288 (37.2%)	1128 (35.5%)	1076 (33.3%)	
Dietary energy intake (kcal/day)	1970 (1517, 2550)	1935 (1423, 2506)	1973 (1528, 2578)	2004 (1567, 2563)	**< 0.001**
Medical condition					
Hypertension (%)	4042 (30.7%)	1424 (30.5%)	1295 (31.4%)	1323 (30.2%)	0.665
Diabetes (%)	1422 (9.2%)	500 (9.6%)	468 (9.3%)	454 (8.9%)	0.702
Laboratory data					
White blood cell count (1000 cells/μL)	7.0 (5.8, 8.4)	7.3 (6.0, 8.8)	6.9 (5.7, 8.3)	6.9 (5.6, 8.3)	**< 0.001**
Lymphocyte percent (%)	30.4 (8.1)	30.2 (8.2)	30.5 (7.9)	30.5 (8.2)	0.575
Mean cell volume (fL)	89.4 (86.4, 92.4)	89.4 (86.5, 92.4)	89.1 (86.2, 92.1)	89.8 (86.6, 92.6)	**0.024**
Red cell distribution width (%)	12.9 (12.3, 13.5)	12.8 (12.3, 13.5)	12.9 (12.3, 13.5)	12.9 (12.3, 13.5)	0.707
Albumin (g/L)	42.1 (3.4)	42.2 (3.5)	42.0 (3.3)	42.2 (3.5)	0.167
Glucose (mmol/L)	5.1 (4.7, 5.6)	5.1 (4.7, 5.6)	5.1 (4.7, 5.6)	5.1 (4.7, 5.6)	0.668
Alkaline phosphatase (U/L)	67 (55, 82)	68 (56, 82)	67 (55, 82)	66 (53, 82)	0.052
Creatinine (μmol/L)	74 (64, 88)	76 (64, 89)	74 (64, 88)	73 (64, 86)	**0.044**
C‐reactive protein (mg/dL)	0.18 (0.08, 0.41)	0.20 (0.08, 0.43)	0.17 (0.07, 0.41)	0.17 (0.07, 0.40)	**0.038**

*Note:* PhenoAge was estimated on the basis of chronological age and nine routine blood biomarkers: albumin, alkaline phosphatase, creatinine, glucose, C‐reactive protein, lymphocyte percentage, mean cell volume, red cell distribution width, and white blood cell count. PhenoAge acceleration represented the residual value from the linear regression model of PhenoAge against chronological age. Lymphocyte percentage and albumin were presented as weighted means with standard deviations (SDs). Other continuous variables were presented as weighted medians with interquartile ranges (IQRs). Categorical variables were presented as unweighted counts with weighted proportions (%). Bold values indicate statistically significant results (*p* < 0.05).

Abbreviations: EGCG, epigallocatechin gallate; PIR, poverty‐to‐income ratio.

**FIGURE 1:**
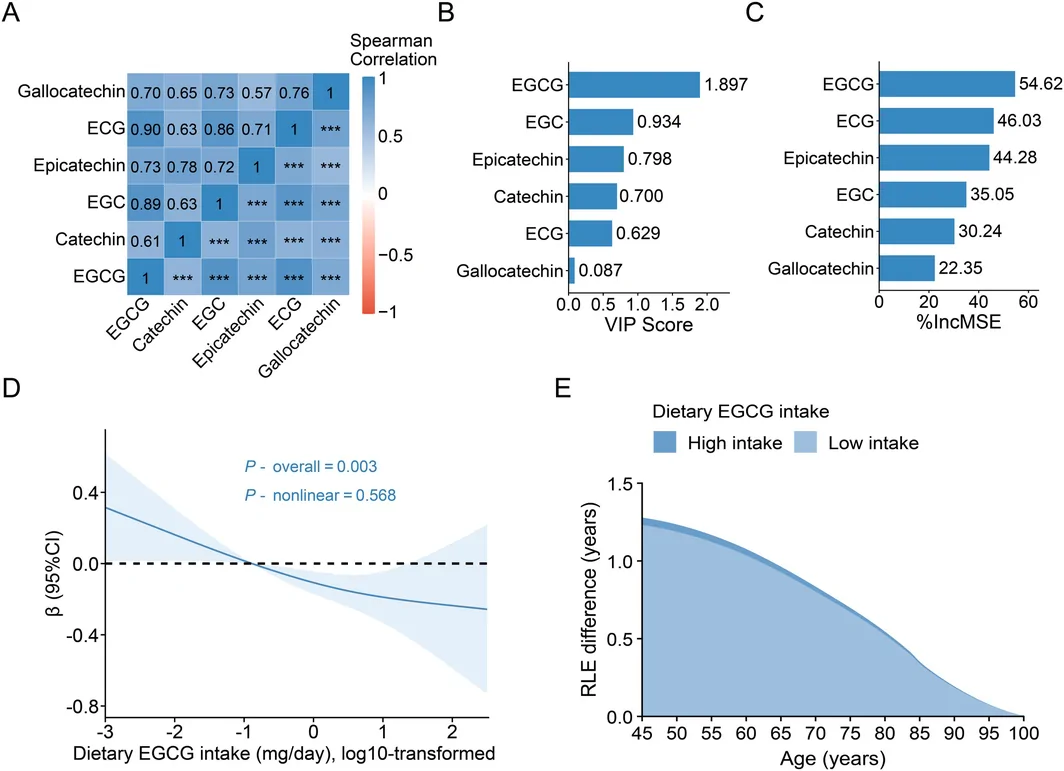
Dietary EGCG intake is associated with lower PhenoAge acceleration and higher estimated residual life expectancy. (A) Spearman correlations among individual dietary catechins; coefficients and significance are shown. (B, C) Importance of dietary catechins in predicting PhenoAge acceleration, assessed by partial least squares regression using variable importance in projection (VIP) scores (B) and by random forest regression using the percentage increase in mean squared error (%IncMSE; C). Higher VIP scores indicate a greater relative contribution to the PLS model. %IncMSE denotes the percentage increase in out‐of‐bag mean squared error after predictor permutation, with larger values indicating greater relative predictive importance. (D) Restricted cubic spline for the association between log_10_‐transformed EGCG intake and PhenoAge acceleration. The *β* coefficient indicates the change in PhenoAge acceleration per one‐unit increase in log_10_‐transformed EGCG intake; solid lines and shaded areas represent estimated associations and 95% confidence intervals, respectively. (E) Estimated differences in residual life expectancy (RLE) associated with low‐ and high‐EGCG intake relative to zero intake. Models were adjusted for age, sex, race, poverty‐to‐income ratio (PIR), smoking, alcohol consumption, physical activity, energy intake, hypertension, and diabetes. ECG, epicatechin gallate; EGC, epigallocatechin; EGCG, epigallocatechin gallate.

### 2.2 Dietary EGCG Intake Is Inversely Associated With PhenoAge Acceleration

Overall, 35.1% of participants were EGCG non‐consumers, and the median EGCG intake in the total population was 0.16 mg/day (IQR: 0.00–28.80). Tea‐based foods and beverages accounted for 99.55% of estimated EGCG intake, with black tea, tea of unspecified type, and green tea contributing 41.00%, 40.18%, and 18.03%, respectively (Table S1, Figure S1). As shown in Table 1, compared with non‐consumers, participants in the higher EGCG intake groups were older, more likely to be female and non‐Hispanic White, and had a higher poverty‐to‐income ratio (PIR) (*p* < 0.05). They had lower PhenoAge acceleration and prevalence of accelerated aging (*p* < 0.001). EGCG consumers were more likely to smoke, less likely to consume alcohol, more frequently engaged in moderate physical activity, and had higher dietary energy intake (*p* < 0.05). White blood cell count, creatinine, and C‐reactive protein were lower in those with higher EGCG intake (*p* < 0.05).

Restricted cubic spline analyses showed a linear inverse association between EGCG intake (log_10_‐transformed) and PhenoAge acceleration in the fully adjusted model (overall *p* = 0.003; nonlinearity *p* = 0.568; Figure 1D). Weighted multivariable linear regression showed that low and high EGCG intake were associated with differences of −0.53 (95% CI −0.99 to −0.08) and −0.62 (95% CI −1.06 to −0.17) years in PhenoAge acceleration relative to non‐consumers, respectively (*p* < 0.05; Table 2, Model 3). This inverse association was not modified by age, sex, race, PIR, smoking, alcohol consumption, physical activity, hypertension, or diabetes (all *p* for interaction > 0.05; Figure S2). Sensitivity analyses supported these inverse associations after excluding participants with energy intakes in the lowest or highest 2.5% (Table S2), applying propensity score weighting (Table S3; Figure S3), and modeling EGCG intake as a continuous log_10_‐transformed variable (Table S4).

**TABLE 2:** Weighted multiple linear regression for the association between dietary EGCG intake categories and PhenoAge acceleration in the overall population (*N* = 10,977).

Variables	Model 1: *β* (95% CI)	*p*	Model 2: *β* (95% CI)	*p*	Model 3: *β* (95% CI)	*p*
Categories of EGCG intake (mg/day)						
Zero (0)	Reference	—	Reference	—	Reference	—
Low (≤ 1.42)	−0.91 (−1.36, −0.45)	**< 0.001**	−0.73 (−1.16, −0.29)	**0.002**	−0.53 (−0.99, −0.08)	**0.023**
High (> 1.42)	−1.10 (−1.58, −0.62)	**< 0.001**	−0.78 (−1.26, −0.30)	**0.002**	−0.62 (−1.06, −0.17)	**0.008**

*Note:* Model 1 was adjusted for age, sex, race, and poverty‐to‐income ratio. Model 2 was adjusted for Model 1 covariates plus smoking status, alcohol consumption, physical activity, and dietary energy intake. Model 3 was adjusted for Model 2 covariates plus hypertension and diabetes. Bold values indicate statistically significant results (*p* < 0.05).

Abbreviations: CI, confidence interval; EGCG, epigallocatechin gallate.

### 2.3 Dietary EGCG Intake Is Associated With Lower Mortality Risk

Over a mean follow‐up of 7.9 years, 1276 deaths (11.6%) occurred. Weighted Cox regression showed an inverse association between EGCG intake and all‐cause mortality. Compared with non‐consumers, fully adjusted hazard ratios (HRs) were 0.83 (95% CI 0.69–0.99) and 0.82 (95% CI 0.68–0.99) for the low‐ and high‐intake groups, respectively (*p* < 0.05; Table S5, Model 3). EGCG consumers had higher estimated residual life expectancy, defined as the area under the estimated survival curve from age 45 to 100 (Figure 1E). At age 45, the estimated differences in residual life expectancy relative to non‐consumers were 1.22 (95% CI 0.38–2.11) and 1.27 (95% CI 0.39–2.25) years for the low‐ and high‐intake groups, respectively. Accelerated‐aging subgroups showed similar patterns (Figure S4).

### 2.4 EGCG Supplementation Extends *C. elegans* Lifespan and Health Span

To further examine population‐level associations linking EGCG to delayed aging and reduced mortality, we evaluated EGCG effects on *C. elegans* lifespan and health span. EGCG significantly extended lifespan at 100–300 μM, with 200 μM yielding the maximal effect (10.73% increase; *p* < 0.001; Figure 2A,B; Table S6), making it the optimal concentration for subsequent assays. This extension persisted on heat‐killed *Escherichia coli* OP50 (*p* < 0.001; Figure S5, Table S6), indicating independence from bacterial metabolism. In aged worms, EGCG improved health span, as indicated by attenuated age‐related declines in pharyngeal pumping and body bending and reduced lipofuscin accumulation, an autofluorescent marker of aging (all *p* < 0.001; Figure 2C–E).

**FIGURE 2:**
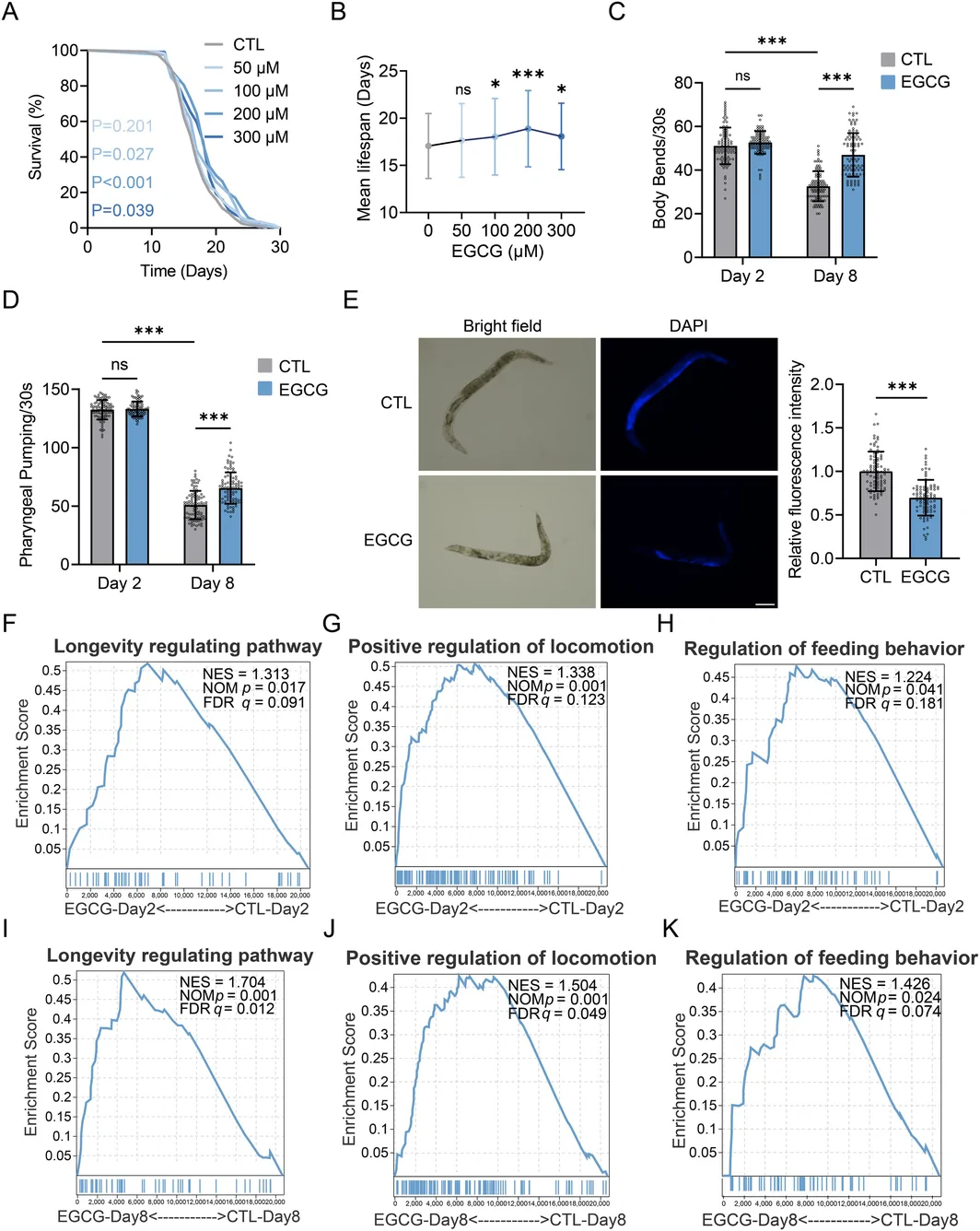
EGCG extends lifespan and health span in *C. elegans* . (A, B) Survival curves and mean lifespan of worms treated with EGCG (0–300 μM). *p* values were calculated using the log‐rank test. (C–E) Body‐bending frequency (C) and pharyngeal pumping rate (D) measured on adult Days 2 and 8, and lipofuscin fluorescence (E) measured on adult Day 8. Scale bar, 100 μm. Data are presented as means ± SD from 90 worms across three independent experiments. One‐way ANOVA with Tukey's test was used for (C, D), and Student's *t*‐test was used for (E). (F–K) GSEA of EGCG‐treated versus control worms on adult Day 2 (F–H) and Day 8 (I–K). Genes were ranked by differential expression between EGCG‐treated and control worms; the enrichment curve shows the running enrichment score, and vertical black bars mark genes in the corresponding gene set. Normalized enrichment score (NES) values, nominal *p* (NOM *p*) values, and false discovery rates (FDR *q*) are annotated; significance was defined as |NES| > 1, nominal *p* < 0.05, and FDR *q* < 0.25. CTL, control; EGCG, epigallocatechin gallate; NS, not significant; *p < 0.05; **p < 0.01; ***p < 0.001.

Gene set enrichment analysis (GSEA) on RNA‐sequencing data from young (Day 2) and aged (Day 8) worms revealed that EGCG significantly enriched the “longevity regulating pathway”, “positive regulation of locomotion”, and “regulation of feeding behavior” at both stages compared to controls (normalized enrichment score [NES]: 1.224–1.704; nominal *p* < 0.05, false discovery rate [FDR] < 0.25; Figure 2F–K). These positive enrichment patterns indicate that EGCG is associated with coordinated modulation of transcriptional programs related to longevity, locomotion, and feeding behavior. Together, these transcriptional and phenotypic findings support beneficial effects of EGCG on *C. elegans* lifespan and health span.

### 2.5 Peroxisome Activation Emerges as a Central Mechanism of EGCG‐Mediated Anti‐Aging

To define baseline aging‐associated transcriptional changes, we compared untreated worms at Days 2 and 8 with those at Day 0. Aging‐downregulated genes were commonly enriched in terms related to locomotion, feeding behavior, lipid homeostasis, fatty acid β‐oxidation, and the peroxisome (Figure S6). To determine whether EGCG could reverse these changes, we analyzed EGCG‐treated worms at Days 2 and 8. On Day 2, EGCG induced 3158 differentially expressed genes (DEGs; Figure S7A), including 3130 upregulated genes enriched in lifespan, locomotion, lipid metabolism, and organelles such as the peroxisome (Figure 3A). On Day 8, 464 DEGs were identified (Figure S7B), including 447 upregulated genes enriched in lipid metabolism, energy homeostasis, and protein modification (Figure 3B). Trend analysis revealed eight distinct expression profiles across both stages (Figure 3C,D). Among them, Profile 3 was the most biologically informative and conserved pattern, as it captured genes whose expression progressively declined in controls but was restored by EGCG treatment, with this reversal remaining statistically significant at both time points (*p* < 0.05). Therefore, the 1130 genes shared in this profile between stages represent a conserved EGCG‐responsive gene set linked to a younger expression state. Kyoto Encyclopedia of Genes and Genomes (KEGG) analysis identified the peroxisome pathway as the top‐enriched pathway among these 1130 genes, supported by Gene Ontology (GO) enrichment of peroxisome terms (*Q*‐value < 0.05; Figure 3E,F). GSEA independently confirmed peroxisome pathway activation at both stages (Day 2, NES = 1.300; Day 8, NES = 1.417; FDR *q* < 0.25; Figure 3G), alongside broadly restored peroxisomal gene expression relative to age‐matched controls (Figure S8).

**FIGURE 3:**
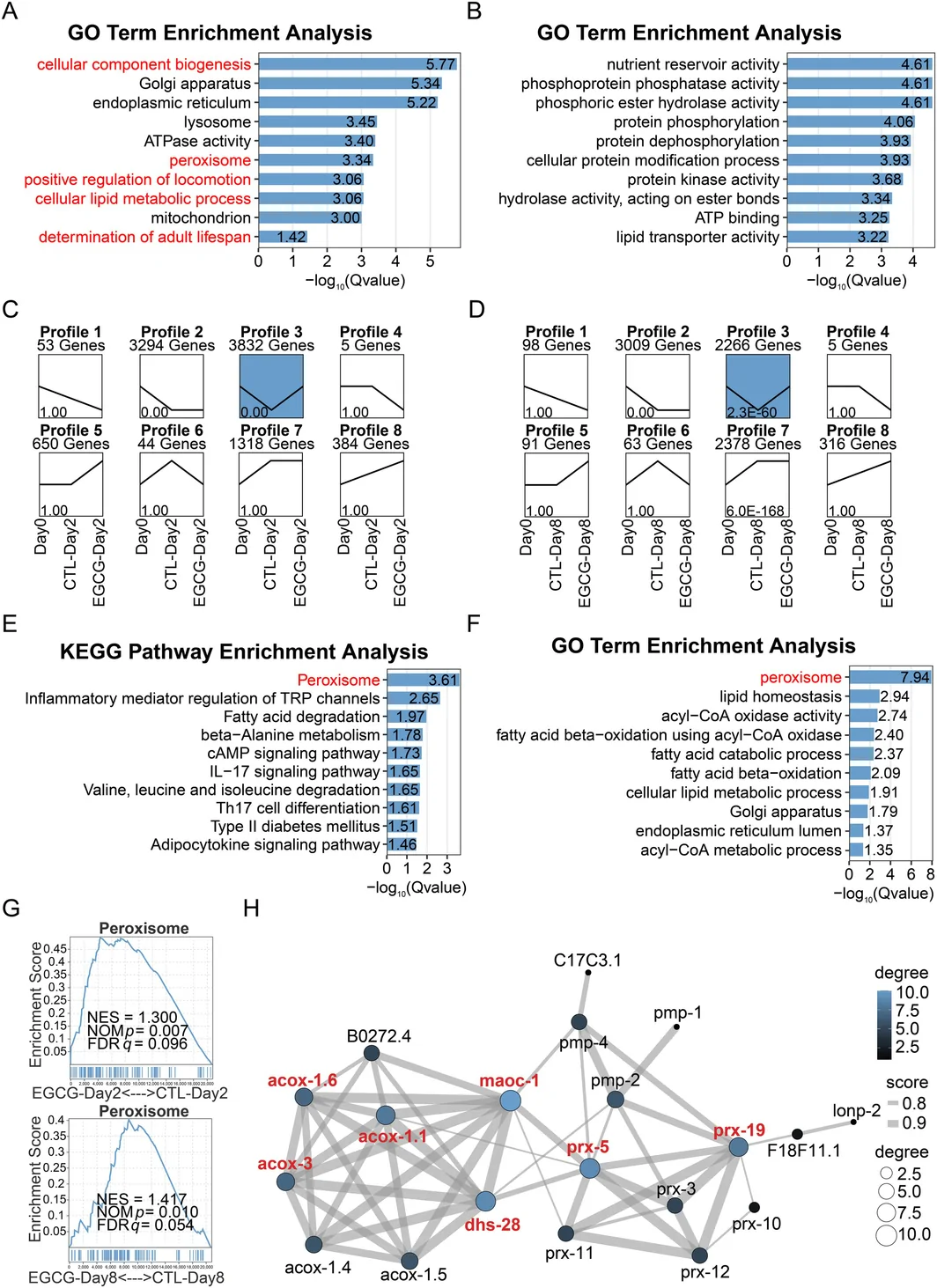
Transcriptomic analyses implicate peroxisome‐related pathways in EGCG‐mediated anti‐aging effects. (A, B) GO enrichment analysis of differentially expressed genes (DEGs) between EGCG‐treated and age‐matched control worms on adult Day 2 (A) and Day 8 (B). (C, D) Line plots showing gene expression patterns associated with age advancement and EGCG treatment on adult Day 2 (C) and Day 8 (D). Profiles with *p* < 0.05 were considered significant. (E, F) KEGG pathway and GO enrichment analyses of 1130 genes consistently reversed by EGCG, identified as the overlap of Profile 3 genes on Day 2 and Day 8. (G) GSEA of the KEGG “Peroxisome” gene set on adult Day 2 and Day 8. (H) High‐confidence protein–protein interaction network of peroxisome‐related DEGs on adult Day 2, defined as DEGs annotated to the GO term “peroxisome” (27 input genes; 20 connected genes). Key hub genes are labeled. (A, B, E, F) Representative significantly enriched GO terms and KEGG pathways are shown (*Q*‐value < 0.05). CTL, control; EGCG, epigallocatechin gallate.

Given the strong peroxisome‐related enrichment at Day 2 (Figure 3A), we performed protein–protein interaction (PPI)‐based prioritization of Day 2 peroxisome‐annotated DEGs (*n* = 27). This yielded a 20‐gene network with two modules corresponding to peroxisomal fatty acid β‐oxidation and peroxisome biogenesis (Figure 3H). Key hub genes included *maoc‐1*, *dhs‐28*, *acox‐1.1*, *acox‐3*, *acox‐1.6*, *prx‐5*, and *prx‐19*, indicating coordinated activation of peroxisome‐related pathways by EGCG during aging.

### 2.6 EGCG Delays Aging by Promoting Peroxisome Biogenesis

The peroxisome biogenesis module in the PPI network (Figure 3H) prompted us to test whether EGCG promotes peroxisome biogenesis to support longevity. EGCG significantly increased peroxisome abundance in *vha‐6p*::mRFP‐PTS1 reporter worms (*p* < 0.001; Figure 4A) and upregulated multiple peroxisome biogenesis genes, including *prx‐1*, *prx‐2*, *prx‐3*, *prx‐5*, *prx‐6*, *prx‐11*, *prx‐12*, *prx‐13*, *prx‐14*, and *prx‐19* (all *p* < 0.05; Figure 4B). Among these, the hub genes *prx‐5* and *prx‐19* were selected for protein‐level validation, and EGCG elevated PRX‐5 and PRX‐19 protein levels in their respective mNeonGreen reporter strains (*p* < 0.001; Figure 4C,D).

**FIGURE 4:**
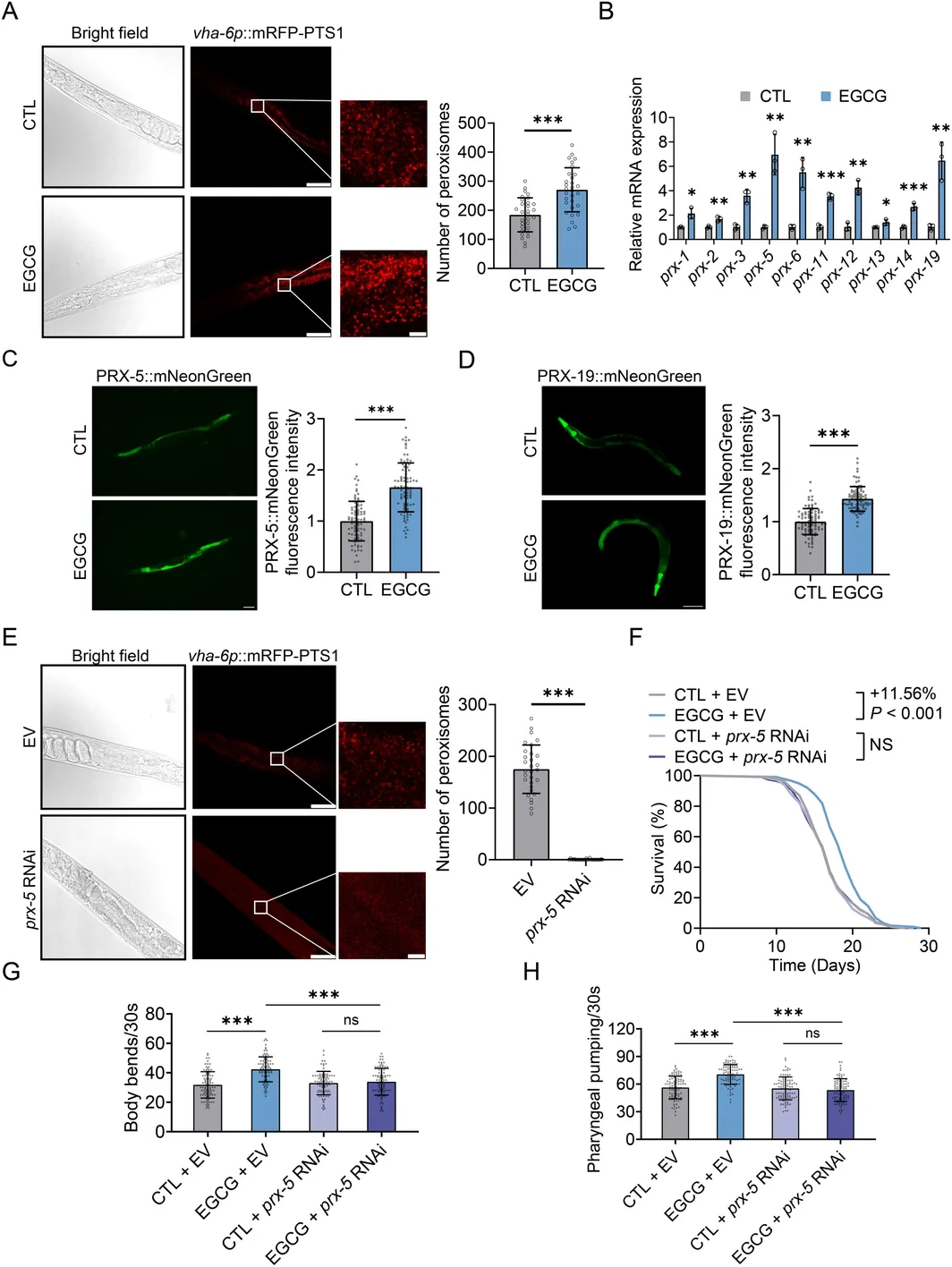
EGCG extends lifespan and health span by enhancing peroxisome biogenesis. (A) Representative images and quantification of intestinal peroxisomes in worms expressing *vha‐6p*::mRFP‐PTS1. Scale bars, 50 μm (overview) and 5 μm (inset). (B) Relative mRNA levels of peroxisome biogenesis‐related genes. (C, D) Representative images and quantification of PRX‐5::mNeonGreen (C) and PRX‐19::mNeonGreen (D) fluorescence. Scale bar, 100 μm. (E) Representative images and quantification of intestinal peroxisomes in *vha‐6p*::mRFP‐PTS1 worms under the indicated RNAi conditions. Scale bars, 50 μm (overview) and 5 μm (inset). (F) Survival curves of worms under the indicated EGCG treatment and RNAi conditions. Survival assays were initiated on adult Day 0; statistical significance was determined using the log‐rank test. (G, H) Body‐bending frequency (G) and pharyngeal pumping rate (H) on adult Day 8 under the indicated EGCG treatment and RNAi conditions. Data in (A–E, G, H) are presented as means ± SD from three independent experiments, with 30 worms for (A, E) and 90 worms for (C, D, G, H). Student's *t*‐tests were used for (A–E), and one‐way ANOVA with Tukey's test was used for (G, H). CTL, control; EGCG, epigallocatechin gallate; EV, empty vector; NS, not significant; *p < 0.05; **p < 0.01; ***p < 0.001.

To test whether peroxisome biogenesis is required, we knocked down *prx‐5*, which mediates PTS1‐dependent matrix protein import monitored by the *vha‐6p*::mRFP‐PTS1 reporter. *prx‐5* knockdown changed the punctate mRFP‐PTS1 signal to a diffuse cytosolic pattern, consistent with impaired PTS1‐dependent matrix protein import and reduced peroxisome abundance (*p* < 0.001; Figure 4E). Crucially, *prx‐5* knockdown abolished EGCG‐mediated longevity and maintenance of pharyngeal and locomotor performance during aging (Figure 4F–H, Table S6). Thus, peroxisome biogenesis is essential for EGCG‐induced lifespan extension and health span improvement.

### 2.7 EGCG Delays Aging by Enhancing Peroxisomal Fatty Acid β‐Oxidation

Enrichment and PPI analyses implicated peroxisomal fatty acid β‐oxidation in the EGCG response (Figure 3F,H). Consistently, EGCG upregulated peroxisomal β‐oxidation genes, including *acox‐1.1*, *acox‐1.2*, *acox‐1.3*, *acox‐1.4*, *acox‐1.5*, *acox‐1.6*, *acox‐3*, *maoc‐1*, and *dhs‐28* (all *p* < 0.05; Figure 5A). *acox‐1.2* and *acox‐3* were prioritized based on evidence linking *acox‐1.2* to lifespan regulation and *acox‐3* to lipid‐droplet homeostasis, a longevity‐associated process involving the coordinated regulation of lipid droplets and peroxisomes (Papsdorf et al. 2023; Wang et al. 2022; Wu et al. 2023). EGCG increased *acox‐1.2* transcriptional activity and ACOX‐3::mNeonGreen protein levels (both *p* < 0.001; Figure 5B,C), confirming activation at both the transcript and protein levels.

**FIGURE 5:**
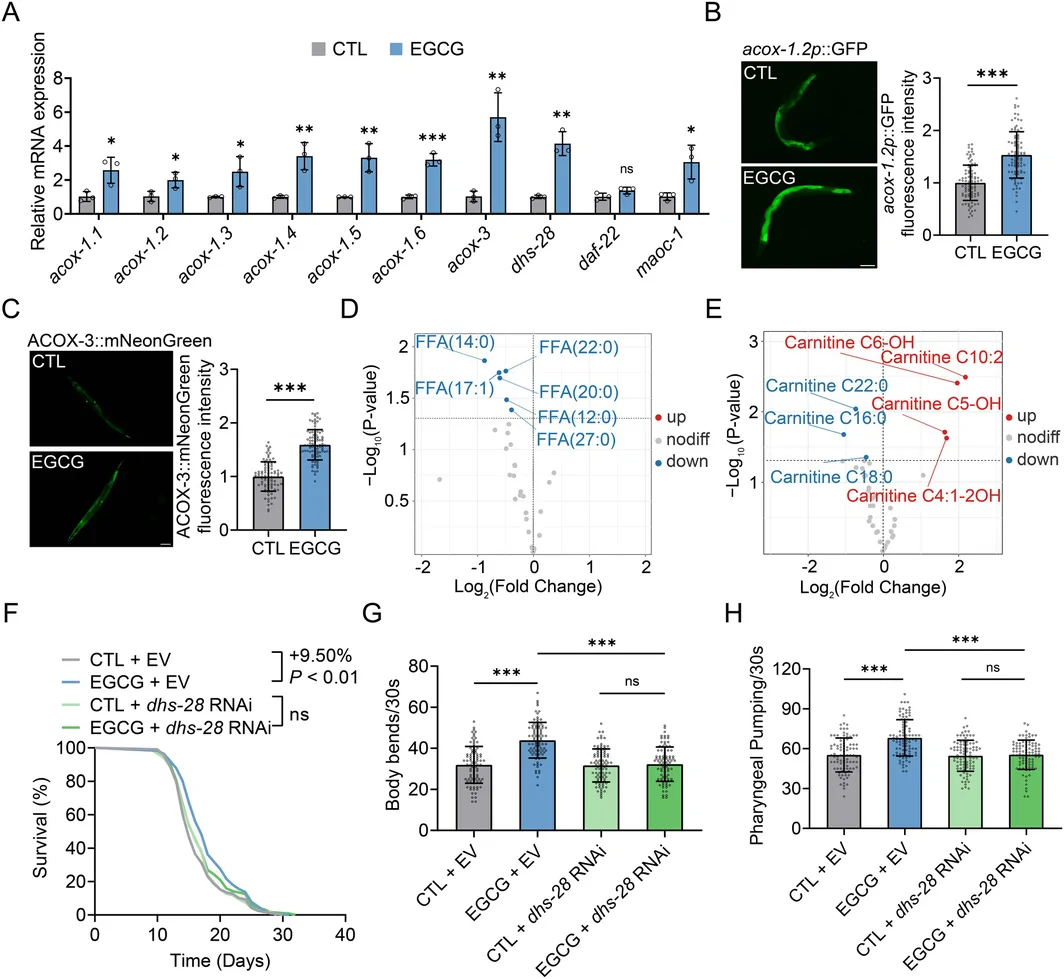
EGCG enhances peroxisomal fatty acid β‐oxidation to extend lifespan and improve health span. (A) Relative mRNA levels of peroxisomal fatty acid β‐oxidation‐related genes. (B, C) Representative images and quantification of *acox‐1.2p*::GFP (B) and ACOX‐3::mNeonGreen (C) fluorescence. Scale bar, 100 μm. (D, E) Volcano plots of differentially abundant free fatty acids (D) and acylcarnitines (E) in EGCG‐treated versus control worms. Significantly altered lipids were defined by VIP > 1 and *p* < 0.05. (F) Survival curves of worms under the indicated EGCG treatment and RNAi conditions. Survival assays were initiated on adult Day 0; statistical significance was determined using the log‐rank test. (G, H) Body‐bending frequency (G) and pharyngeal pumping rate (H) on adult Day 8 under the indicated EGCG treatment and RNAi conditions. Data in (A–C, G, H) are presented as means ± SD from three independent experiments, with 90 worms for (B, C, G, H). Student's *t*‐tests were used for (A–C), and one‐way ANOVA with Tukey's test was used for (G, H). CTL, control; EGCG, epigallocatechin gallate; EV, empty vector; NS, not significant; *p < 0.05; **p < 0.01; ***p < 0.001.

Targeted lipidomics revealed that EGCG reduced VLCFAs (C22:0 and C27:0), long‐chain fatty acids, very‐long‐chain acylcarnitine C22:0, and long‐chain acylcarnitines, while increasing short‐ and medium‐chain acylcarnitines (all *p* < 0.05; Figure 5D,E), consistent with enhanced peroxisomal β‐oxidation and flux toward shorter‐chain intermediates.

Since ACOX generates H_2_O_2_ as a byproduct, we measured endogenous H_2_O_2_ levels. EGCG induced an early, transient increase in H_2_O_2_, followed by increased *ctl‐1*, *ctl‐2*, and *ctl‐3* expression and catalase activity, and reduced H_2_O_2_ levels later in life (Figure S9A–E). Furthermore, *acox‐1.2* knockdown reduced H_2_O_2_ levels and both the EGCG‐induced H_2_O_2_ increase and lifespan extension, as did N‐acetylcysteine (NAC) co‐treatment (Figure S9F–J; Table S6). To further test the requirement for peroxisomal β‐oxidation, *dhs‐28* knockdown also abolished EGCG‐mediated lifespan and health span benefits (Figure 5F–H; Table S6). We next examined the effects of EGCG in worms with alterations in established longevity pathways. EGCG extended lifespan in dietary‐restricted *eat‐2*(*ad1116*) mutants and autophagy‐impaired *bec‐1* RNAi worms, but not in *aak‐2*(*ok524*), *skn‐1*(*zu135*), or *isp‐1*(*qm150*) mutants, implicating AAK‐2/AMPK and SKN‐1/NRF2 signaling and mitochondrial function in its longevity effects (Figure S10).

### 2.8 EGCG Alleviates Doxorubicin‐Induced Senescence Through Peroxisome‐Related Pathways in AML12 Hepatocytes

To test whether the peroxisome‐related anti‐aging mechanism extends to mammalian cells, we treated doxorubicin‐induced senescent AML12 hepatocytes with EGCG. Doxorubicin, a DNA‐damaging agent commonly used to induce cellular senescence (Zhang et al. 2025), increased senescence‐associated β‐galactosidase (SA‐β‐gal)‐positive cells and p21/p16 protein levels, whereas EGCG attenuated these senescence‐associated phenotypes (Figure 6A,B).

**FIGURE 6:**
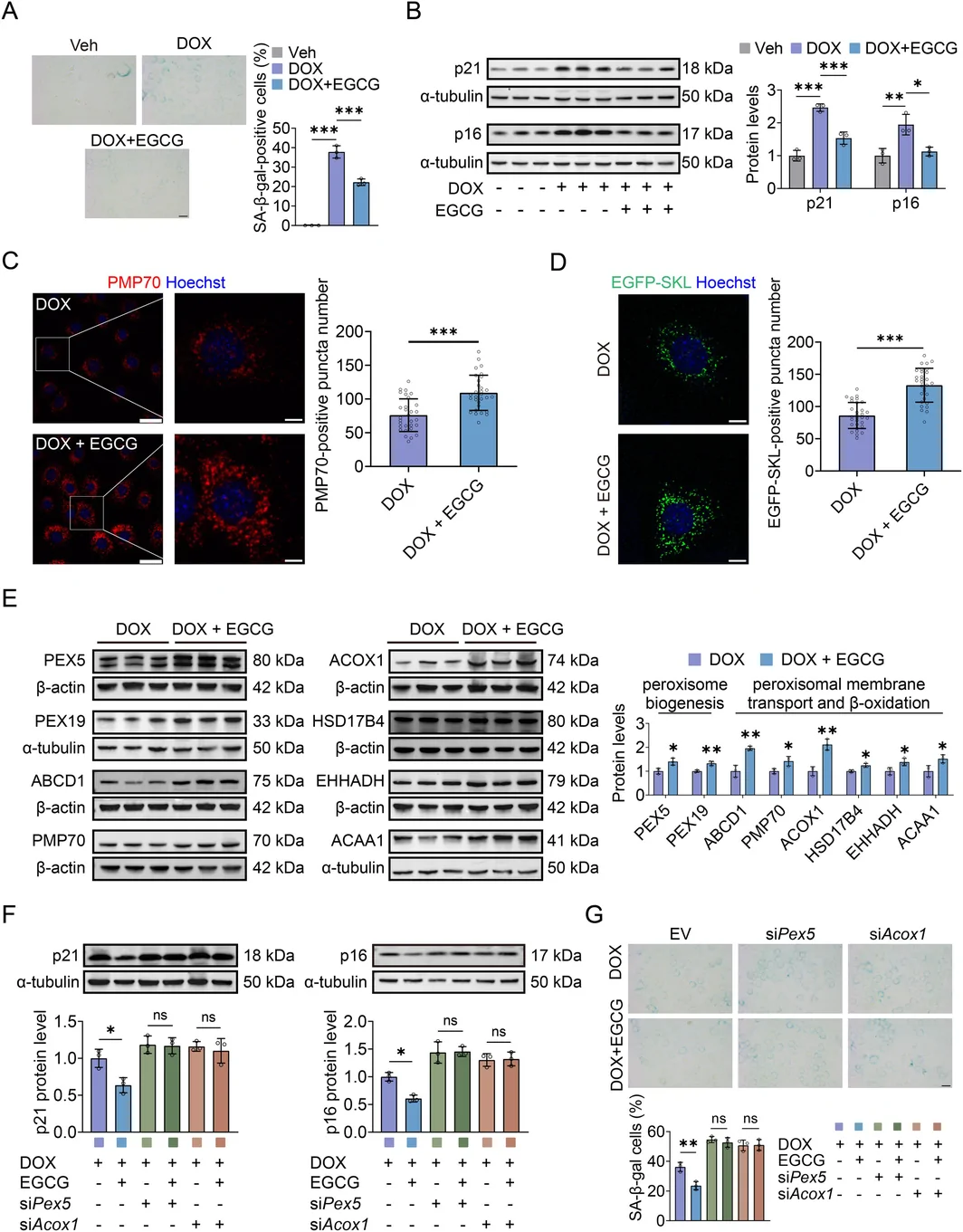
EGCG alleviates doxorubicin‐induced senescence by activating peroxisome biogenesis and peroxisomal fatty acid β‐oxidation in AML12 cells. (A) Representative images and quantification of SA‐β‐gal‐positive cells. Scale bar, 50 μm. (B) Representative immunoblots and quantification of p21 and p16. (C, D) Representative images and quantification of PMP70‐positive puncta (C) and EGFP‐SKL‐positive puncta (D). Scale bars, 50 μm (overview) and 10 μm (inset). (E) Representative immunoblots and quantification of peroxisome‐related proteins. (F) Representative immunoblots and quantification of p21 and p16 under the indicated combinations of DOX, EGCG, si*Pex5*, and si*Acox1*. (G) Representative images and quantification of SA‐β‐gal‐positive cells under the indicated combinations of DOX, EGCG, si*Pex5*, and si*Acox1*. Scale bar, 50 μm. All data are presented as means ± SD from three independent experiments, with three biological replicates for (A, B, E–G) and 30 cells for (C, D). One‐way ANOVA with Tukey's test was used for (A, B, F, G), and Student's *t*‐tests were used for (C–E). DOX, doxorubicin; EGCG, epigallocatechin gallate; NS, not significant; SA‐β‐gal, senescence‐associated β‐galactosidase; Veh, vehicle; *p < 0.05; **p < 0.01; ***p < 0.001.

PMP70 immunofluorescence and EGFP‐SKL imaging showed that EGCG increased peroxisome abundance (*p* < 0.001; Figure 6C,D). EGCG increased the levels of PEX5, PEX19, ABCD1, PMP70, and the β‐oxidation enzymes ACOX1, HSD17B4, EHHADH, and ACAA1 (*p* < 0.05; Figure 6E), indicating enhanced peroxisome biogenesis and peroxisomal fatty acid β‐oxidation capacity.

To determine whether these peroxisome‐related pathways were required for the anti‐senescent effect of EGCG, siRNA‐mediated knockdown of *Pex5* or *Acox1* was performed. Knockdown of either gene diminished the ability of EGCG to suppress p21 and p16 protein expression and reduce SA‐β‐gal positivity (Figure 6F,G), suggesting that peroxisome biogenesis and β‐oxidation contribute to the anti‐senescent effect of EGCG. Finally, EGCG‐associated human genes from the Comparative Toxicogenomics Database were enriched for “Longevity regulating pathway—multiple species”, “Peroxisome”, and “fatty acid metabolic process” terms (corrected *p* < 0.01; Figure S11), supporting the relevance of peroxisome‐ and longevity‐related pathways to EGCG responses across species.

## 3 Discussion

Integrating population‐based and mechanistic evidence, this study shows that dietary EGCG intake is associated with lower PhenoAge acceleration and all‐cause mortality risk in humans, and EGCG extends lifespan and health span in worms and alleviates doxorubicin‐induced senescence in AML12 hepatocytes. As cellular senescence contributes to organismal aging through tissue dysfunction and senescent‐cell accumulation (Cai et al. 2022), EGCG‐mediated senescence attenuation in AML12 cells complements its benefits in *C. elegans* . Notably, our data identify the peroxisome as an important mediator of EGCG‐promoted healthy aging, whereby EGCG promotes both peroxisome biogenesis and peroxisomal fatty acid β‐oxidation, contributing to delayed aging phenotypes.

In the nationally representative NHANES population, higher dietary EGCG intake was associated with lower PhenoAge acceleration, lower all‐cause mortality risk, and higher estimated residual life expectancy. Our analysis identified that tea, predominantly black and green tea, was the main dietary EGCG source, indicating tea consumption as a practical EGCG source. This finding is consistent with reports linking tea consumption to slower biological aging and reduced mortality (Inoue‐Choi et al. 2022; Xiang et al. 2024). We prioritized PhenoAge acceleration, given that it captures biological age deviations from chronological age and is associated with mortality risk and life expectancy (Chen, Wu, et al. 2024; Feng et al. 2024; Liu et al. 2018). In our study, higher EGCG intake was associated with lower white blood cell count, creatinine, and C‐reactive protein, although absolute differences were small and values remained within expected clinical ranges. These patterns may indicate subtle differences in inflammation or kidney function, aspects of which are captured by PhenoAge and may be improved by EGCG, as previously reported (Niu et al. 2013; Ramesh et al. 2010), potentially contributing to the associations with biological aging and survival. Notably, EGCG intake was highly right‐skewed (median, approximately 0.16 mg/day; 95th percentile, 143.91 mg/day; 99th percentile, 332.61 mg/day). Upper‐tail values are broadly comparable to ranges reported in a European Union population assessment (EFSA ANS Panel (EFSA Panel on Food Additives and Nutrient Sources added to Food) et al. 2018). Thus, despite generally low dietary exposure in NHANES, the associations may reflect cumulative chronic low‐dose or indirect effects, including gut microenvironment modulation. Although experimental EGCG doses cannot be directly translated to human intakes given major interspecies differences in absorption and metabolism, the concordance between epidemiologic and experimental findings supports EGCG as a candidate dietary compound associated with healthy aging.

Our data indicate that EGCG increases peroxisome biogenesis and abundance, and that peroxisome biogenesis is required for EGCG‐induced lifespan and health span extension in *C. elegans* and senescence attenuation in AML12 hepatocytes. This is consistent with prior work showing that increasing peroxisome biogenesis or abundance promotes longevity (Papsdorf et al. 2023; Wu et al. 2023). Aging is often accompanied by reduced peroxisomal protein abundance and impaired matrix‐protein import, changes thought to contribute to dysregulated lipid homeostasis (Guo et al. 2025; Vercaemst et al. 2026). Conversely, we found that EGCG reverses the age‐associated decline in peroxisome‐related gene expression at the transcriptome level, supporting a model in which EGCG helps maintain metabolic homeostasis by preserving or restoring peroxisomal function. Notably, in our study, knockdown of *prx‐5*, the *C. elegans* ortholog of mammalian *PEX5*, abolished EGCG‐mediated lifespan extension, whereas *Pex5* knockdown in AML12 hepatocytes diminished the ability of EGCG to suppress doxorubicin‐induced senescence. As previously reported, disruption of peroxisome‐biogenesis genes can attenuate cellular responses to EGCG (Akla et al. 2026). Collectively, these findings establish peroxisome biogenesis as a critical determinant of EGCG responsiveness and its anti‐aging effects.

We next investigated the effect of EGCG on peroxisomal fatty acid β‐oxidation. In *C. elegans* , EGCG increased *acox* gene family expression and protein levels of ACOX‐3, a homolog of mammalian ACOX1 (Wang et al. 2022), the rate‐limiting enzyme in peroxisomal fatty‐acid β‐oxidation. In AML12 hepatocytes, EGCG increased the protein abundance of ACOX1, HSD17B4, EHHADH, and ACAA1. Notably, similar upregulation has been observed in the livers of long‐lived endocrine mutant mice (Elmansi and Miller 2023). Conversely, ACOX1 inhibition shifts the fatty‐acid profile of young rat brains toward an aged‐like state (Rafiei et al. 2023), suggesting that reinforcing peroxisomal β‐oxidation could oppose age‐related lipid changes. Functionally, the loss of EGCG‐induced lifespan extension after *dhs‐28* or *acox‐1.2* knockdown in *C. elegans* , together with the weakened anti‐senescent effect after *Acox1* knockdown in AML12 hepatocytes, supports the relevance of peroxisomal β‐oxidation to EGCG responsiveness, consistent with previous evidence that *acox‐1.2* is required for α‐ketobutyrate‐induced lifespan extension in *C. elegans* (Wu et al. 2023). At the metabolite level, our targeted lipidomics further indicated increased peroxisomal β‐oxidation flux, characterized by reduced VLCFAs and increased shorter‐chain acylcarnitine intermediates. Given that aging is associated with impaired VLCFA processing and VLCFA accumulation (Guo et al. 2025), which in turn promote oxidative stress, inflammatory responses, and mitochondrial dysfunction (Kim and Bai 2022; Wanders et al. 2023), our findings support that EGCG enhances peroxisomal VLCFA processing, thereby limiting VLCFA accumulation and its associated lipotoxic, oxidative, and pro‐inflammatory consequences, ultimately contributing to health span and lifespan extension.

Building on the lipidomic evidence, it is reasonable to consider whether these metabolic changes may influence mitochondrial fatty acid oxidation. Peroxisomes and mitochondria cooperate in lipid catabolism, with peroxisomal VLCFA shortening generating intermediates that can undergo further oxidation in mitochondria. Consistent with this framework, our lipidomic data revealed reduced VLCFAs alongside increased medium‐ and short‐chain acylcarnitines, a pattern compatible with enhanced peroxisomal chain shortening and increased availability of downstream β‐oxidation substrates (Wanders et al. 2023). Although we did not directly measure inter‐organelle metabolite transfer, these findings place EGCG‐induced peroxisomal activation within a broader peroxisome–mitochondria lipid‐oxidation axis that has been associated with longevity‐promoting states (Elmansi and Miller 2023; Weir et al. 2017).

The lack of additional lifespan extension by EGCG in *aak‐2*, *skn‐1*, and *isp‐1* mutants may place peroxisomal remodeling within an interconnected network involving AAK‐2/AMPK and SKN‐1/NRF2 signaling and mitochondrial function. Peroxisomes and mitochondria cooperate in fatty acid oxidation (Wanders et al. 2023), whereas AAK‐2/AMPK promotes longevity through functional coordination between these organelles to enhance fatty acid oxidation (Weir et al. 2017). Enhanced peroxisomal β‐oxidation also promotes longevity in *C. elegans* through signaling mediated by SKN‐1 (Wu et al. 2023). These observations provide a potential framework for understanding how EGCG‐induced peroxisomal remodeling engages established longevity pathways, although their directionality and hierarchy remain unresolved.

EGCG treatment was accompanied by an early, transient increase in endogenous H_2_O_2_, followed by increased catalase activity and lower H_2_O_2_ levels at later time points. These temporal changes suggest that EGCG elicits a transient redox response rather than sustained H_2_O_2_ accumulation. Consistently, EGCG induces a transient ROS increase followed by antioxidant activation in *C. elegans* (Xiong et al. 2018), and enhanced peroxisomal β‐oxidation increases H_2_O_2_ production (Wu et al. 2023). In our study, *acox‐1.2* knockdown and NAC treatment affected both the early H_2_O_2_ increase and EGCG‐mediated lifespan extension. The biological relevance of this H_2_O_2_ response to EGCG‐associated lifespan extension remains unclear, given the complex and context‐dependent relationship between ROS and lifespan regulation (Karagianni and Bazopoulou 2024).

The EGCG‐driven increase in peroxisome abundance may provide an organellar basis for enhanced β‐oxidation, although the causal relationship remains unestablished. An upstream regulatory axis could coordinate these linked processes. Previous studies indicate that EGCG activates peroxisome proliferator‐activated receptor α (PPARα) and increases transcription of PPARα‐responsive genes involved in peroxisomal and mitochondrial fatty acid β‐oxidation (Ding et al. 2020; Sun et al. 2025). Moreover, PPARα and its *C. elegans* ortholog NHR‐49 are linked to peroxisome biogenesis and lipid oxidation (Jiang et al. 2022; Tsai et al. 2024). Together, these findings suggest that EGCG may engage an NHR‐49/PPARα‐centered program to couple these responses, thereby delaying aging; this hypothesis requires genetic and mechanistic testing.

Taken together, a major strength is the integrative framework combining mechanistic experiments in *C. elegans* and AML12 hepatocytes with population‐based NHANES analyses, linking population‐level associations to findings spanning organismal lifespan and health span and mammalian cellular senescence. Collective evidence from transcriptomic profiling, reporter imaging, complementary molecular analyses, targeted lipidomics, and loss‐of‐function experiments supports peroxisome biogenesis and fatty acid β‐oxidation as functionally important components of the anti‐aging response to EGCG. Several limitations should be noted. First, EGCG estimates from two self‐reported 24‐h recalls may misrepresent habitual intake owing to daily variation, recall bias, and uncertainty in database‐derived EGCG values, potentially attenuating associations. Meanwhile, categorizing right‐skewed intake may obscure dose–response patterns, although results were generally consistent using log_10_‐transformed intake. Moreover, residual confounding remains possible. Prospective studies with repeated assessments and validated catechin biomarkers are needed. Second, findings from *C. elegans* and AML12 cells should not be directly extrapolated to human aging. Although Comparative Toxicogenomics Database enrichment results suggest potential relevance of peroxisome‐ and longevity‐related pathways to EGCG responses in humans, animal and human studies are needed to establish whether the peroxisome‐centered mechanism is conserved in vivo and contributes to health outcomes. Additionally, FUDR may affect lifespan‐related outcomes or interact with EGCG or RNAi interventions; extension to FUDR‐free conditions remains to be tested. Third, EGCG absorption and metabolism in *C. elegans* remain unclear. Lifespan extension with live or dead bacteria suggests bacterial viability is not required, but indirect effects through the intestinal environment or host–microbe interactions cannot be excluded. EGCG can be biotransformed by intestinal microorganisms (Zhang et al. 2024), but whether intact EGCG or worm‐ or microbe‐derived metabolites mediate these effects requires metabolomic and isotope‐tracing studies. Finally, upstream links between EGCG and the peroxisomal program, including potential NHR‐49/PPARα involvement, remain undefined.

In conclusion, EGCG intake was inversely associated with human biological aging metrics, while experimental findings support a peroxisome‐centered mechanism for its longevity and anti‐senescent effects. These findings refine current understanding of EGCG‐associated anti‐aging biology and suggest that modulation of peroxisomal pathways by natural compounds may represent a feasible strategy to promote healthy aging.

## 4 Methods

### 4.1 Study Population

We analyzed data from NHANES 2007–2008, 2009–2010, and 2017–2018. Protocols were approved by the National Center for Health Statistics Ethics Review Board (#2005‐06 for 2007–2010; #2011‐17 and #2018‐01 for 2017–2018), and all participants provided written informed consent. Adults aged ≥ 20 years were included. Participants missing either 24‐h dietary recall data for catechin estimation, biomarkers required for PhenoAge calculation, or covariates were excluded. The cross‐sectional sample comprised 10,977 adults; 10,964 participants with linked mortality data were included in mortality analyses (Figure S12).

### 4.2 Assessment of Dietary Catechin Intake

Intakes of EGCG, epicatechin, epigallocatechin, epicatechin gallate, catechin, and gallocatechin were obtained from the United States Department of Agriculture Flavonoid Intake Data Files and averaged across two valid 24‐h dietary recalls. Details of data derivation and dietary source attribution of EGCG are provided in the Supplementary Methods. Given that 35.1% of participants reported no EGCG intake (0 mg/day) and intake among consumers was markedly right‐skewed, consumers were dichotomized at the median into low (≤ 1.42 mg/day) and high (> 1.42 mg/day) intake groups, yielding three approximately equal‐sized groups.

### 4.3 PhenoAge and PhenoAge Acceleration

Biological aging was assessed using PhenoAge, derived from chronological age and nine routine blood biomarkers: albumin, alkaline phosphatase, creatinine, glucose, C‐reactive protein, lymphocyte percentage, mean cell volume, red blood cell distribution width, and white blood cell count (Levine et al. 2018; Liu et al. 2018). PhenoAge acceleration was the residual from a linear regression of PhenoAge on chronological age. Positive and negative values denote accelerated and non‐accelerated biological aging, respectively (Chen, Wu, et al. 2024; Liu et al. 2018).

### 4.4 All‐Cause Mortality

Mortality was ascertained via the National Death Index. Follow‐up duration spanned from the baseline Mobile Examination Center examination until death or December 31, 2019, whichever occurred first.

### 4.5 Covariates

Covariates included age, sex, race, PIR, smoking, alcohol consumption, physical activity, dietary energy intake, hypertension, and diabetes. PIR categories were < 1.3, 1.3–3.5, and > 3.5 (Li et al. 2025). Smoking was defined as ≥ 100 lifetime cigarettes, and alcohol consumption as ≥ 12 drinks per year (Ao et al. 2025; Zheng et al. 2025). Physical activity was categorized as low (< 600), moderate (≥ 600 to < 3000), or vigorous (≥ 3000 metabolic equivalent of task [MET]‐min/week) (Cai et al. 2024). Dietary energy intake was averaged across two 24‐h recalls. Hypertension and diabetes were based on self‐reported physician diagnosis.

### 4.6 Worm Strains and Maintenance

*C. elegans* strains were obtained from the Caenorhabditis Genetics Center (CGC) or generated by SunyBiotech (Fuzhou, China). Strain and transgenic construct details are provided in Table S7 and the Supplementary Methods. Worms were maintained on nematode growth medium (NGM) plates seeded with *E. coli* OP50 at 20°C.

### 4.7 Cell Culture

AML12 is a nontransformed mouse hepatocyte cell line retaining differentiated hepatocyte features, including peroxisomes (Wu et al. 1994). AML12 cells (Procell Life Science & Technology, China) were maintained in AML12 Cell Complete Medium (Procell) at 37°C and 5% CO_2_ in a humidified incubator.

### 4.8 Pharmacological Treatments

For worm experiments, EGCG (≥ 95%; Sigma‐Aldrich, USA) and NAC (Sigma‐Aldrich) were dissolved in sterile water as 10‐ and 250‐mM stock solutions, respectively. Stocks were added to NGM at 20 μL/mL, yielding final concentrations of 200 μM EGCG and 5 mM NAC, respectively; control plates received the equivalent volume of sterile water. In all *C. elegans* experiments, worms were exposed to 50 μM fluorodeoxyuridine (FUDR; Sigma‐Aldrich) on NGM plates from Day 0 adulthood onward to suppress progeny development.

For cell experiments, doxorubicin (Sigma‐Aldrich) was dissolved in DMSO to prepare a 100 μM stock solution. Senescence was induced with 100 nM doxorubicin for 24 h, followed by incubation with or without 50 μM EGCG for 48 h.

### 4.9 Lifespan Assays

Synchronized Day‐0 adults were exposed to EGCG or vehicle on NGM plates seeded with OP50 or RNAi bacteria. Survival was scored daily; worms unresponsive to gentle prodding were considered dead. Sample sizes for all lifespan assays are provided in Table S6.

### 4.10 Health Span Assays

Health span was assessed by pharyngeal pumping, body bending, and lipofuscin autofluorescence. Terminal pharyngeal bulb contractions on NGM plates seeded with OP50 or RNAi bacteria and body bends in M9 buffer (42.3 mM Na_2_HPO_4_, 22.0 mM KH_2_PO_4_, 85.6 mM NaCl, and 1.0 mM MgSO_4_) were counted for 30 s under a stereomicroscope. For lipofuscin quantification, worms anesthetized with 5 mM levamisole hydrochloride were imaged using a Nikon ECLIPSE Ci‐L upright fluorescence microscope (Nikon, Japan) with a 10× objective and DAPI filter set (excitation, 340–390 nm; emission, 420–470 nm). Each worm was manually delineated as a region of interest in Fiji (ImageJ v1.54p), and mean fluorescence intensity was quantified after subtracting background fluorescence from an adjacent worm‐free region.

### 4.11 SA‐β‐Gal Staining

SA‐β‐gal activity was assessed using a Senescence β‐Galactosidase Staining Kit (Beyotime, China). Cells were washed with PBS, fixed with the kit‐provided fixation solution for 15 min at room temperature, and incubated overnight at 37°C without CO_2_ in freshly prepared kit‐based X‐Gal solution. Blue SA‐β‐gal‐positive cells were imaged using a Leica DMi8 inverted microscope (Leica, Germany) with a 20× objective and quantified as a percentage of all cells.

### 4.12 RNA Interference

RNA interference (RNAi) was performed by feeding synchronized L1 larvae with HT115(DE3) bacteria expressing gene‐specific double‐stranded RNA (dsRNA) on NGM plates containing ampicillin (100 μg/mL) and isopropyl β‐D‐1‐thiogalactopyranoside (IPTG; 1 mM). RNAi clones targeting *acox‐1.1*, *acox‐1.2*, *acox‐1.3*, *acox‐1.4*, and *acox‐1.5* were constructed by SunyBiotech; those targeting *acox‐1.6*, *acox‐3*, *prx‐5*, *dhs‐28*, and *bec‐1* were obtained from the Vidal library. Empty‐vector HT115(DE3) was the control. Worms were exposed to RNAi bacteria from L1 onward, with EGCG or vehicle treatment initiated on adult Day 0. Knockdown efficiency was verified by quantitative real‐time PCR (qRT–PCR). Clone construction and bacterial strain details are in the Supplementary Methods and Table S8.

### 4.13 Transfection of AML12 Cells

AML12 cells in confocal dishes were transfected with EGFP‐SKL plasmid (1 μg). Cells in six‐well plates were transfected with siRNAs targeting *Pex5* or *Acox1* or with non‐targeting control siRNA (NC), at 50 nM using Lipofectamine 3000 (Thermo Fisher Scientific). Knockdown was assessed by qRT‐PCR and Western blotting. Plasmid and siRNAs were obtained from HanBio Biotechnology (Shanghai, China); details are in the Supplementary Methods and Table S9.

### 4.14 Immunofluorescence Staining

AML12 cells were fixed with 4% paraformaldehyde for 10 min, permeabilized with 0.1% Triton X‐100 for 15 min, and blocked with 4% normal goat serum for 1 h at room temperature. Cells were incubated with rabbit anti‐PMP70 antibody (Abmart; 1:100) overnight at 4°C, followed by Alexa Fluor 594‐conjugated goat anti‐rabbit IgG (Thermo Fisher Scientific; 1:200) for 1 h in the dark and DAPI (1 μg/mL) for 10 min.

### 4.15 Microscopy and Fluorescence Quantification

Synchronized Day‐0 adult transgenic *C. elegans* expressing *acox‐1.2p*::GFP, ACOX‐3::mNeonGreen, PRX‐5::mNeonGreen, PRX‐19::mNeonGreen, or *vha‐6p*::mRFP‐PTS1 were treated with EGCG or vehicle for 48 h, anesthetized with 5 mM levamisole hydrochloride, and mounted on 2% agarose pads. GFP and mNeonGreen signals were imaged on a Nikon ECLIPSE Ci‐L microscope using a 10× or 20× objective and a FITC filter set (excitation, 455–495 nm, emission, 505–555 nm). Fluorescence intensity was quantified in Fiji as described for lipofuscin in Section 4.10.

Intestinal peroxisomes in worms were visualized with *vha‐6p*::mRFP‐PTS1, whereas cellular peroxisomes were detected using EGFP‐SKL or PMP70 immunostaining. mRFP‐PTS1, EGFP‐SKL, PMP70, and DAPI signals were acquired using a Leica SP8 confocal microscope with a 40× objective and excitation wavelengths of 561, 488, 594, and 405 nm, respectively. Images were analyzed in Fiji. mRFP‐PTS1‐positive puncta were counted within a 25 × 25 μm^2^ anterior intestine region (Int1/Int2 cells); EGFP‐SKL‐ and PMP70‐positive puncta were counted per cell. Identical acquisition and analysis settings were used across groups.

### 4.16 RNA Extraction and qRT–PCR

Total RNA was extracted from approximately 200 worms per biological replicate using the SimplyP Total RNA Extraction Kit (Bioer Technology, China) and reverse‐transcribed using Plus All‐in‐one 1st Strand cDNA Synthesis SuperMix (Novoprotein, China). qRT–PCR was performed using Universal Fast SYBR qPCR SuperMix (Novoprotein) on a QuantStudio 5 System (Thermo Fisher Scientific) under the following conditions: 95°C for 30 s, followed by 40 cycles at 95°C for 5 s and 60°C for 15 s. Relative expression levels were normalized to *act‐1*. Primer sequences are listed in Table S10.

### 4.17 Western Blotting

Cellular proteins were extracted in RIPA buffer, quantified by bicinchoninic acid assay, separated by 4%–20% SDS‐PAGE, and transferred to PVDF membranes. Membranes were blocked in 5% non‐fat milk/TBST for 1 h, incubated with primary antibodies overnight at 4°C, and HRP‐conjugated secondary antibodies for 1 h at room temperature. Bands were detected by enhanced chemiluminescence using an e‐BLOT Touch Imager XLi and quantified in Fiji with β‐actin and α‐tubulin as loading controls. Antibodies are listed in Table S11.

### 4.18 RNA Sequencing and Bioinformatic Analysis

Synchronized Day‐0 adults were collected before treatment (Day 0) or after treatment with EGCG or vehicle for 2 or 8 days (approximately 5000 worms per biological replicate; three biological replicates per group, Figure S13). Total RNA was extracted, and libraries were sequenced on an Illumina NovaSeq X Plus system. Differential expression analysis was performed using DESeq2, with DEGs defined as those with a FDR < 0.05 and |log_2_(fold change)| > 1. Functional enrichment, gene set enrichment, expression pattern, and PPI network analyses are detailed in the Supplementary Methods.

### 4.19 Widely Targeted Quantitative Lipidomic Analysis

Synchronized Day‐0 adults were treated with EGCG or vehicle until Day 2 (approximately 10,000 worms per biological replicate; six replicates per group). Lipidomic profiling used ultra‐performance liquid chromatography–tandem mass spectrometry. Differential lipids were defined by VIP > 1 and *p* < 0.05. Details are provided in the Supplementary Methods.

### 4.20 Statistical Analysis

NHANES analyses were conducted in R v4.4.3, accounting for the complex survey design and sampling weights. Spearman correlations assessed intercorrelations among individual dietary catechins, and PLS and random forest regressions prioritized catechins associated with PhenoAge acceleration. Approximately normally distributed continuous variables are reported as weighted means (standard deviation, SD) and compared using weighted analysis of variance (ANOVA); non‐normally distributed continuous variables are reported as weighted medians (IQR) and compared using the weighted Kruskal–Wallis test; categorical variables are reported as unweighted counts with weighted percentages and compared using the weighted chi‐squared test.

Dose–response associations between log_10_‐transformed EGCG intake and PhenoAge acceleration were assessed using weighted restricted cubic splines, with non‐linearity tested by likelihood ratio tests (Crainiceanu and Ruppert 2004). Weighted multivariable linear regression assessed associations between categorized EGCG intake and PhenoAge acceleration. Subgroup analyses utilizing the fully adjusted model (excluding the stratification variable) assessed effect modification. Sensitivity analyses excluded participants with extreme energy intake (lowest or highest 2.5%), applied propensity score weighting to balance covariates, and modeled log_10_‐transformed EGCG intake as a continuous variable. Weighted Cox proportional hazards models assessed associations between categorized EGCG intake and all‐cause mortality, with follow‐up time as the time scale and death as the event. Residual life expectancy at age 45 was estimated as the area under the survival curve from ages 45 to 100, with between‐category area differences representing estimated differences in residual life expectancy (Chen, Wu, et al. 2024).

*C. elegans* and AML12 cell experiments were analyzed using GraphPad Prism version 10 (GraphPad Software, USA). Parametric tests assumed normally distributed data with equal variances. Two‐group comparisons were performed using two‐tailed unpaired Student's *t*‐tests, whereas comparisons among three or more groups were performed using one‐way ANOVA followed by Tukey's post hoc test. Survival was assessed using Kaplan–Meier curves and log‐rank tests. Unless otherwise specified, all tests were two‐sided, and *p* < 0.05 was considered statistically significant.

## Author Contributions

Yin Yuan and Pengli Zhu conceived and designed the study and supervised the project. Yanfang Yang and Feng Huang collected database data. Xiaoming Huang, Yanfang Yang, and Feng Huang performed statistical analyses, interpreted findings, and prepared figures; Xiaoming Huang and Feng Huang conducted bioinformatics analyses. Xiaoming Huang, Wenwen Lin, and Qiaowei Li performed experiments and managed experimental data. Xiaoming Huang, Yanfang Yang, and Feng Huang drafted the manuscript; Yin Yuan and Pengli Zhu reviewed and revised the manuscript. All authors approved the final manuscript.

## Funding

This work was supported by the Science and Technology Innovation Joint Major Project of Fujian Province (grant number 2023Y9285); Natural Science Foundation of Fujian Province (grant number 2022J05209); the Joint Funds for the innovation of science and Technology, Fujian province (grant number 2025Y9029); and Major Scientific Research Project of Fujian Provincial Health Commission (grant number 2022ZD01006).

## Conflicts of Interest

The authors declare no conflicts of interest.

## Supporting information


**Figure S1:** Contribution of major food and beverage categories to total estimated dietary EGCG intake.
**Figure S2:** Subgroup analyses of the association of dietary EGCG intake categories with PhenoAge acceleration.
**Figure S3:** Absolute standardized mean differences (ASMDs) for covariates in the unweighted sample and after survey and propensity score weighting.
**Figure S4:** Estimated differences in residual life expectancy by dietary EGCG intake category, stratified by accelerated aging status.
**Figure S5:** Kaplan–Meier survival curves of worms treated with EGCG (200 μM) or vehicle under heat‐killed OP50 feeding.
**Figure S6:** Gene Ontology (GO) enrichment analysis of aging‐associated downregulated genes in untreated control worms.
**Figure S7:** Volcano plots of EGCG‐induced differentially expressed genes (DEGs) in young and aged adult worms.
**Figure S8:** Gene expression heatmap for the KEGG “peroxisome” pathway across time points and treatments.
**Figure S9:** EGCG treatment is associated with altered hydrogen peroxide homeostasis and lifespan extension.
**Figure S10:** AAK‐2/AMPK, SKN‐1/NRF2, and mitochondrial function contribute to EGCG‐associated lifespan extension.
**Figure S11:** Gene Ontology (GO) and KEGG pathway enrichment results of EGCG‐associated human genes retrieved from the Comparative Toxicogenomics Database.
**Figure S12:** Flowchart of participant selection in the National Health and Nutrition Examination Survey (NHANES).
**Figure S13:** Transcriptomic sampling scheme in *Caenorhabditis elegans* .
**Table S1:** Top 20 U.S. Department of Agriculture (USDA) food codes ranked by contribution to total estimated dietary EGCG intake.
**Table S2:** Weighted multiple linear regression for the association of dietary EGCG intake categories with PhenoAge acceleration after excluding participants with extreme energy intake (*N* = 10,428).
**Table S3:** Survey and propensity score‐weighted multiple linear regression for the association of dietary EGCG intake categories with PhenoAge acceleration in the overall population (*N* = 10,977).
**Table S4:** Weighted multiple linear regression for the association between log_10_‐transformed dietary EGCG intake and PhenoAge acceleration in the overall population (*N* = 10,977).
**Table S5:** Weighted multivariable Cox proportional hazards models for the association of dietary EGCG intake categories with all‐cause mortality in the overall population (*N* = 10,964).
**Table S6:** Summary of lifespan experiments.
**Table S7:** *C. elegans* strains used in this study.
**Table S8:** Information on RNAi feeding clones obtained from the Vidal library and used in this study.
**Table S9:** siRNA sequences used in AML12 cells.
**Table S10:** Primers used for quantitative RT–PCR.
**Table S11:** Antibodies used for western blot analysis.

## Data Availability

The NHANES datasets used in the present study are publicly available through the official website at https://www.cdc.gov/nchs/nhanes/. Any additional data generated or analyzed during this study can be obtained from the corresponding authors upon reasonable request.

## References

[acel70795-bib-0001] Akla, N. , A. Boudah , T. Bertomeu , A. Chatr‐Aryamontri , M. Desjarlais , and B. Annabi . 2026. “CRISPR‐Based Chemogenomic Profiling Reveals Redox Vulnerabilities to Epigallocatechin‐3‐Gallate and Green Tea Polyphenol Extract.” Redox Biology 90: 104047. 10.1016/j.redox.2026.104047. PMC12860261 41581373

[acel70795-bib-0002] Ao, Y. , F. Xiao , J. Qiu , et al. 2025. “The Association Between the Uric Acid‐to‐High‐Density Lipoprotein Cholesterol Ratio and Osteoarthritis Risk in U.S. Adults: A Cross‐Sectional Study Based on NHANES 1999–2016.” Lipids in Health and Disease 24: 191. 10.1186/s12944-025-02618-2. PMC12107929 40420274

[acel70795-bib-0003] Cai, C. , S. Zhu , M. Qin , et al. 2024. “Long‐Term Exposure to PM2.5 Chemical Constituents and Diabesity: Evidence From a Multi‐Center Cohort Study in China.” Lancet Regional Health—Western Pacific 47: 101100. 10.1016/j.lanwpc.2024.101100. PMC11179652 38881803

[acel70795-bib-0004] Cai, Y. , W. Song , J. Li , et al. 2022. “The Landscape of Aging.” Science China Life Sciences 65: 2354–2454. 10.1007/s11427-022-2161-3. PMC9446657 36066811

[acel70795-bib-0005] Campos, J. C. , L. H. Marchesi Bozi , B. Krum , et al. 2023. “Exercise Preserves Physical Fitness During Aging Through AMPK and Mitochondrial Dynamics.” Proceedings of the National Academy of Sciences of the United States of America 120: e2204750120. 10.1073/pnas.2204750120. PMC9926278 36595699

[acel70795-bib-0006] Chen, J.‐C. , R. Wang , and C.‐C. Wei . 2024. “Anti‐Aging Effects of Dietary Phytochemicals: From *Caenorhabditis elegans* , *Drosophila melanogaster* , Rodents to Clinical Studies.” Critical Reviews in Food Science and Nutrition 64: 5958–5983. 10.1080/10408398.2022.2160961. 36597655

[acel70795-bib-0007] Chen, L. , B. Wu , L. Mo , et al. 2024. “Associations Between Biological Ageing and the Risk of, Genetic Susceptibility to, and Life Expectancy Associated With Rheumatoid Arthritis: A Secondary Analysis of Two Observational Studies.” Lancet Healthy Longevity 5: e45–e55. 10.1016/S2666-7568(23)00220-9. 38081205

[acel70795-bib-0008] Crainiceanu, C. M. , and D. Ruppert . 2004. “Likelihood Ratio Tests for Goodness‐Of‐Fit of a Nonlinear Regression Model.” Journal of Multivariate Analysis 91: 35–52. 10.1016/j.jmva.2004.04.008.

[acel70795-bib-0009] Ding, H. , Y. Li , W. Li , et al. 2020. “Epigallocatechin‐3‐Gallate Activates the AMP‐Activated Protein Kinase Signaling Pathway to Reduce Lipid Accumulation in Canine Hepatocytes.” Journal of Cellular Physiology 236: 405–416. 10.1002/jcp.29869. 32572960

[acel70795-bib-0010] EFSA ANS Panel (EFSA Panel on Food Additives and Nutrient Sources added to Food) , M. Younes , P. Aggett , et al. 2018. “Scientific Opinion on the Safety of Green Tea Catechins.” EFSA Journal 16: 5239. 10.2903/j.efsa.2018.5239. PMC7009618 32625874

[acel70795-bib-0011] Elmansi, A. M. , and R. A. Miller . 2023. “Coordinated Transcriptional Upregulation of Oxidative Metabolism Proteins in Long‐Lived Endocrine Mutant Mice.” GeroScience 45: 2967–2981. 10.1007/s11357-023-00849-8. PMC10643730 37273159

[acel70795-bib-0012] Feng, Y. , H. Lin , H. Tan , and X. Liu . 2024. “Heterogeneity of Aging and Mortality Risk Among Individuals With Hypertension: Insights From Phenotypic Age and Phenotypic Age Acceleration.” Journal of Nutrition, Health & Aging 28: 100203. 10.1016/j.jnha.2024.100203. PMC12877294 38460315

[acel70795-bib-0013] Guo, X. , G. Du , J. Zhou , et al. 2025. “Aging‐Related Peroxisomal Dysregulation Disrupts Intestinal Stem Cell Differentiation Through Alterations of Very Long‐Chain Fatty Acid Oxidation.” PLoS Biology 23: e3003552. 10.1371/journal.pbio.3003552. PMC12716710 41417787

[acel70795-bib-0014] Huang, K. , T. Miao , K. Chang , et al. 2020. “Impaired Peroxisomal Import in *Drosophila* Oenocytes Causes Cardiac Dysfunction by Inducing upd3 as a Peroxikine.” Nature Communications 11: 2943. 10.1038/s41467-020-16781-w. PMC7286907 32523050

[acel70795-bib-0015] Inoue‐Choi, M. , Y. Ramirez , M. C. Cornelis , A. Berrington de González , N. D. Freedman , and E. Loftfield . 2022. “Tea Consumption and All‐Cause and Cause‐Specific Mortality in the UK Biobank: A Prospective Cohort Study.” Annals of Internal Medicine 175: 1201–1211. 10.7326/M22-0041. PMC10623338 36037472

[acel70795-bib-0016] Jiang, S. , M. J. Uddin , X. Yu , et al. 2022. “Peroxisomal Fitness: A Potential Protective Mechanism of Fenofibrate Against High Fat Diet‐Induced Non‐Alcoholic Fatty Liver Disease in Mice.” Diabetes & Metabolism Journal 46: 829–842. 10.4093/dmj.2021.0274. PMC9723204 35746892

[acel70795-bib-0017] Karagianni, C. , and D. Bazopoulou . 2024. “Redox Regulation in Lifespan Determination.” Journal of Biological Chemistry 300: 105761. 10.1016/j.jbc.2024.105761. PMC10965828 38367668

[acel70795-bib-0018] Kim, J. , and H. Bai . 2022. “Peroxisomal Stress Response and Inter‐Organelle Communication in Cellular Homeostasis and Aging.” Antioxidants 11: 192. 10.3390/antiox11020192. PMC8868334 35204075

[acel70795-bib-0019] Kirchweger, B. , J. Zwirchmayr , U. Grienke , and J. M. Rollinger . 2023. “The Role of *Caenorhabditis elegans* in the Discovery of Natural Products for Healthy Aging.” Natural Product Reports 40: 1849–1873. 10.1039/D3NP00021D. 37585263

[acel70795-bib-0020] Levine, M. E. , A. T. Lu , A. Quach , et al. 2018. “An Epigenetic Biomarker of Aging for Lifespan and Healthspan.” Aging 10: 573–591. 10.18632/aging.101414. PMC5940111 29676998

[acel70795-bib-0021] Li, B. , Z. Ming , Y. Wang , et al. 2025. “Supplement‐Driven Iron Overload Accelerates Phenotypic Aging via Inflammatory Biomarkers: Potential Counteraction Through Anti‐Inflammatory or Antioxidant Diets.” Redox Biology 85: 103733. 10.1016/j.redox.2025.103733. PMC12246624 40570516

[acel70795-bib-0022] Liu, Z. , P.‐L. Kuo , S. Horvath , E. Crimmins , L. Ferrucci , and M. Levine . 2018. “A New Aging Measure Captures Morbidity and Mortality Risk Across Diverse Subpopulations From NHANES IV: A Cohort Study.” PLoS Medicine 15: e1002718. 10.1371/journal.pmed.1002718. PMC6312200 30596641

[acel70795-bib-0023] Niu, Y. , L. Na , R. Feng , et al. 2013. “The Phytochemical, EGCG, Extends Lifespan by Reducing Liver and Kidney Function Damage and Improving Age‐Associated Inflammation and Oxidative Stress in Healthy Rats.” Aging Cell 12: 1041–1049. 10.1111/acel.12133. 23834676

[acel70795-bib-0024] Papsdorf, K. , J. W. Miklas , A. Hosseini , et al. 2023. “Lipid Droplets and Peroxisomes Are Co‐Regulated to Drive Lifespan Extension in Response to Mono‐Unsaturated Fatty Acids.” Nature Cell Biology 25: 672–684. 10.1038/s41556-023-01136-6. PMC10185472 37127715

[acel70795-bib-0025] Rafiei, S. , F. Khodagholi , H. Gholami Pourbadie , L. Dargahi , and F. Motamedi . 2023. “Hepatic Acyl CoA Oxidase1 Inhibition Modifies Brain Lipids and Electrical Properties of Dentate Gyrus.” Basic and Clinical Neuroscience 14: 663–674. 10.32598/bcn.2021.3500.1. PMC11016873 38628834

[acel70795-bib-0026] Ramesh, E. , P. Geraldine , and P. A. Thomas . 2010. “Regulatory Effect of Epigallocatechin Gallate on the Expression of C‐Reactive Protein and Other Inflammatory Markers in an Experimental Model of Atherosclerosis.” Chemico‐Biological Interactions 183: 125–132. 10.1016/j.cbi.2009.09.013. 19782057

[acel70795-bib-0027] Sun, J. , C. Yin , Z. Li , et al. 2025. “Natural Compounds Regulating Fatty Acid Oxidation in the Treatment of Diabetic Kidney Disease.” Frontiers in Nutrition 12: 1669557. 10.3389/fnut.2025.1669557. PMC12568393 41170356

[acel70795-bib-0028] Tsai, S.‐H. , Y.‐C. Wu , D. F. Palomino , F. C. Schroeder , and C.‐L. Pan . 2024. “Peripheral Peroxisomal β‐Oxidation Engages Neuronal Serotonin Signaling to Drive Stress‐Induced Aversive Memory in *C. elegans* .” Cell Reports 43: 113996. 10.1016/j.celrep.2024.113996. PMC11087011 38520690

[acel70795-bib-0029] Vercaemst, A. , M. Zhao , R. Chai , C. Lismont , and M. Fransen . 2026. “Peroxisomes in Aging: Guardians of Cellular Resilience and Function.” Cells 15: 254. 10.3390/cells15030254. PMC12897043 41677620

[acel70795-bib-0030] Wanders, R. J. A. , M. Baes , D. Ribeiro , S. Ferdinandusse , and H. R. Waterham . 2023. “The Physiological Functions of Human Peroxisomes.” Physiological Reviews 103: 957–1024. 10.1152/physrev.00051.2021. 35951481

[acel70795-bib-0031] Wang, T. , H. Xu , S. Wu , Y. Guo , G. Zhao , and D. Wang . 2023. “Mechanisms Underlying the Effects of the Green Tea Polyphenol EGCG in Sarcopenia Prevention and Management.” Journal of Agricultural and Food Chemistry 71: 9609–9627. 10.1021/acs.jafc.3c02023. 37316469

[acel70795-bib-0032] Wang, Y. , C. Li , J. Zhang , et al. 2022. “Polyunsaturated Fatty Acids Promote the Rapid Fusion of Lipid Droplets in *Caenorhabditis elegans* .” Journal of Biological Chemistry 298: 102179. 10.1016/j.jbc.2022.102179. PMC9352923 35752365

[acel70795-bib-0033] Weir, H. J. , P. Yao , F. K. Huynh , et al. 2017. “Dietary Restriction and AMPK Increase Lifespan via Mitochondrial Network and Peroxisome Remodeling.” Cell Metabolism 26: 884–896.e5. 10.1016/j.cmet.2017.09.024. PMC5718936 29107506

[acel70795-bib-0034] Wu, J. C. , G. Merlino , and N. Fausto . 1994. “Establishment and Characterization of Differentiated, Nontransformed Hepatocyte Cell Lines Derived From Mice Transgenic for Transforming Growth Factor Alpha.” Proceedings of the National Academy of Sciences of the United States of America 91: 674–678. 10.1073/pnas.91.2.674. PMC43011 7904757

[acel70795-bib-0035] Wu, N. , Y.‐C. Ma , X.‐Q. Gong , et al. 2023. “The Metabolite Alpha‐Ketobutyrate Extends Lifespan by Promoting Peroxisomal Function in *C. elegans* .” Nature Communications 14: 240. 10.1038/s41467-023-35899-1. PMC9842765 36646719

[acel70795-bib-0036] Xiang, Y. , H. Xu , H. Chen , et al. 2024. “Tea Consumption and Attenuation of Biological Aging: A Longitudinal Analysis From Two Cohort Studies.” Lancet Regional Health—Western Pacific 42: 100955. 10.1016/j.lanwpc.2023.100955. PMC10700389 38075587

[acel70795-bib-0037] Xiong, L.‐G. , Y.‐J. Chen , J.‐W. Tong , Y.‐S. Gong , J.‐A. Huang , and Z.‐H. Liu . 2018. “Epigallocatechin‐3‐Gallate Promotes Healthy Lifespan Through Mitohormesis During Early‐to‐Mid Adulthood in *Caenorhabditis elegans* .” Redox Biology 14: 305–315. 10.1016/j.redox.2017.09.019. PMC5635249 28992589

[acel70795-bib-0038] Yuan, H. , Y. Li , F. Ling , et al. 2020. “The Phytochemical Epigallocatechin Gallate Prolongs the Lifespan by Improving Lipid Metabolism, Reducing Inflammation and Oxidative Stress in High‐Fat Diet‐Fed Obese Rats.” Aging Cell 19: e13199. 10.1111/acel.13199. PMC7511879 32729662

[acel70795-bib-0039] Zhang, M. , B. Li , and J. Tian . 2023. “Mitochondrial Targets Exploration of Epigallocatechin Gallate and Theaflavin in Regards to Differences in Stress Protection Under Different Temperatures.” Journal of Nutritional Biochemistry 119: 109400. 10.1016/j.jnutbio.2023.109400. 37271321

[acel70795-bib-0040] Zhang, S. , B. Mao , S. Cui , et al. 2024. “Absorption, Metabolism, Bioactivity, and Biotransformation of Epigallocatechin Gallate.” Critical Reviews in Food Science and Nutrition 64: 6546–6566. 10.1080/10408398.2023.2170972. 36728926

[acel70795-bib-0041] Zhang, Y. , Y. Li , F. Wang , et al. 2025. “Liver‐Bone Organoids Reveal Senescence‐Driven Interorgan Crosstalk.” Bioactive Materials 54: 570–583. 10.1016/j.bioactmat.2025.08.039. PMC12444486 40980505

[acel70795-bib-0042] Zheng, L. , G. Li , J. Cao , Z. Zhao , L. Zhang , and Z. Hou . 2025. “Dietary Copper Intake and Biological Aging Among US Adults, NHANES 2003–2018.” Aging Cell 24: e70272. 10.1111/acel.70272. PMC12686563 41114515

[acel70795-bib-0043] Zhong, J. , J. Fang , Y. Wang , et al. 2024. “Dietary Flavonoid Intake Is Negatively Associated With Accelerating Aging: An American Population‐Based Cross‐Sectional Study.” Nutrition Journal 23: 158. 10.1186/s12937-024-01052-x. PMC11661325 39707398

[acel70795-bib-0044] Zhou, F. , K. Gu , and Y. Zhou . 2023. “Flavonoid Intake Is Associated With Lower All‐Cause and Disease‐Specific Mortality: The National Health and Nutrition Examination Survey 2007–2010 and 2017–2018.” Frontiers in Nutrition 10: 1046998. 10.3389/fnut.2023.1046998. PMC9971007 36866051

