# Supplementary material for: The Dietary Polyphenol Epigallocatechin Gallate Delays Biological Aging by Activating Peroxisome Biogenesis and Peroxisomal Fatty Acid β‐Oxidation
Source: Aging Cell. 2026 Nov 2;25(11):e70795. doi: 10.1111/acel.70795 (PMC13727896; doi:10.1111/acel.70795)
Supplement: Supplementary file 1 [file ACEL-25-e70795-s001.docx]

***Supplementary materials***

**The Dietary Polyphenol Epigallocatechin Gallate Delays Biological Aging by Activating Peroxisome Biogenesis and Peroxisomal Fatty Acid β-Oxidation**

Xiaoming Huang, PhD^1,2,3,4#^, Yanfang Yang, MS^1#^, Feng Huang, MS^1,2,3,4#^, Wenwen Lin, MS^1,2,3,4^, Qiaowei Li, PhD^1,2,3,4^, Yin Yuan, PhD^1,2,3,4*^, Pengli Zhu, MS^1,2,3,4*^

**Figures:**

Figure S1. Contribution of major food and beverage categories to total estimated dietary EGCG intake.

Figure S2. Subgroup analyses of the association of dietary EGCG intake categories with PhenoAge acceleration.

Figure S3. Absolute standardized mean differences (ASMDs) for covariates in the unweighted sample and after survey and propensity score weighting.

Figure S4. Estimated differences in residual life expectancy by dietary EGCG intake category, stratified by accelerated aging status.

Figure S5. Kaplan–Meier survival curves of worms treated with EGCG (200 μM) or vehicle under heat-killed OP50 feeding.

Figure S6. Gene Ontology (GO) enrichment analysis of aging-associated downregulated genes in untreated control worms.

Figure S7. Volcano plots of EGCG-induced differentially expressed genes (DEGs) in young and aged adult worms.

Figure S8. Gene expression heatmap for the KEGG "peroxisome" pathway across time points and treatments.

Figure S9. EGCG treatment is associated with altered hydrogen peroxide homeostasis and lifespan extension.

Figure S10. AAK-2/AMPK, SKN-1/NRF2, and mitochondrial function contribute to EGCG-associated lifespan extension.

Figure S11. Gene Ontology (GO) and KEGG pathway enrichment results of EGCG-associated human genes retrieved from the Comparative Toxicogenomics Database.

Figure S12. Flowchart of participant selection in the National Health and Nutrition Examination Survey (NHANES).

Figure S13. Transcriptomic sampling scheme in *Caenorhabditis elegans*.

**Tables:**

Table S1. Top 20 U.S. Department of Agriculture (USDA) food codes ranked by contribution to total estimated dietary EGCG intake.

Table S2. Weighted multiple linear regression for the association of dietary EGCG intake categories with PhenoAge acceleration after excluding participants with extreme energy intake (N = 10,428).

Table S3. Survey and propensity score-weighted multiple linear regression for the association of dietary EGCG intake categories with PhenoAge acceleration in the overall population (N = 10,977).

Table S4. Weighted multiple linear regression for the association between log_10_-transformed dietary EGCG intake and PhenoAge acceleration in the overall population (N = 10,977).

Table S5. Weighted multivariable Cox proportional hazards models for the association of dietary EGCG intake categories with all-cause mortality in the overall population (N =10,964).

Table S6. Summary of lifespan experiments.

Table S7. *C. elegans* strains used in this study.

Table S8. Information on RNAi feeding clones obtained from the Vidal library and used in this study.

Table S9. siRNA sequences used in AML12 cells.

Table S10. Primers used for quantitative RT–PCR.

Table S11. Antibodies used for western blot analysis.

**Supplementary Methods**

Survey weighting

Assessment of dietary catechin intake

Dietary source attribution of EGCG intake

Partial least squares (PLS) regression

Random forest regression

RNA sequencing and bioinformatic analysis

Widely targeted quantitative lipidomic analysis

Hydrogen peroxide assay

Catalase activity assay

Bicinchoninic acid (BCA) protein assay

EGFP–SKL plasmid construction

Generation of multi-copy integrated fluorescent reporter strains

Construction of RNAi feeding plasmids

**Figure S1. Contribution of major food and beverage categories to total estimated dietary EGCG intake.** Other specified tea includes oolong tea and chai; tea of unspecified type comprises food codes whose main food descriptions did not specify the tea type.

**Figure S2. Subgroup analyses of the association of dietary EGCG intake categories with PhenoAge acceleration.** Models were adjusted for age, sex, race, poverty-to-income ratio (PIR), smoking status, alcohol consumption, physical activity, dietary energy intake, hypertension, and diabetes (excluding the stratifying variable within each analysis). EGCG, epigallocatechin gallate.

**Figure S3. Absolute standardized mean differences (ASMDs) for covariates in the unweighted sample and after survey and propensity score weighting.** ASMDs below 0.10 indicate minimal residual imbalance in measured covariates between the three EGCG intake groups after weighting and are generally considered evidence of adequate covariate balance. PIR, poverty-to-income ratio.

**Figure S4. Estimated differences in residual life expectancy by dietary EGCG intake category, stratified by accelerated aging status.** Estimated differences in residual life expectancy (RLE) associated with low- and high-EGCG intake relative to zero intake among participants with accelerated aging (A) or non-accelerated aging (B). Models were adjusted for age, sex, race, poverty-to-income ratio, smoking, alcohol consumption, physical activity, energy intake, hypertension, and diabetes. EGCG, epigallocatechin gallate.

**Figure S5. Kaplan–Meier survival curves of worms treated with EGCG (200 μM) or vehicle under heat-killed OP50 feeding.** Survival assays were initiated on adult day 0; *P* value was determined using the log-rank test. See Table S6 for detailed lifespan data. CTL, control; EGCG, epigallocatechin gallate.

**Figure S6. Gene Ontology (GO) enrichment analysis of aging-associated downregulated genes in untreated control worms.** (A) Significantly enriched GO terms among genes downregulated at day 2 compared with day 0. (B) Significantly enriched GO terms among genes downregulated at day 8 compared with day 0. All terms shown met the significance threshold of a Q-value < 0.05.

**Figure S7. Volcano plots of EGCG-induced differentially expressed genes (DEGs) in young and aged adult worms.** Volcano plots showed DEGs in EGCG-treated worms compared with age-matched controls at the young adult stage (Day 2, A) and the aged adult stage (Day 8, B). Genes were defined as differentially expressed using the following criteria: false discovery rate (FDR) < 0.05 and |log_2_FC| > 1, where FC denotes fold change. A representative subset of highly responsive genes, defined as those with a log2 fold change greater than 3.32 (more than 10-fold upregulation), is labeled in the volcano plots. These genes are mainly involved in methylation, oxidoreductase and xenobiotic metabolism, glutathione metabolism, transmembrane transport and efflux, transcriptional regulation, and protein catabolism.

**Figure S8. Gene expression heatmap for the KEGG** "**peroxisome**" **pathway across time points and treatments.** Blue denotes low expression and red denotes high expression. Groups were ordered from left to right as Day 0 (baseline control), Day 2 control (CTL-Day2), Day 8 control (CTL-Day8), Day 2 EGCG-treated (EGCG-Day2), and Day 8 EGCG-treated (EGCG-Day8). CTL, control; EGCG, epigallocatechin gallate; KEGG, Kyoto Encyclopedia of Genes and Genomes.

**Figure S9. EGCG treatment is associated with altered hydrogen peroxide homeostasis and lifespan extension.** (A–E) Time-course analyses of H_2_O_2_ homeostasis-related parameters in the indicated groups at adult days 0, 2, 4, 6, 8, and 10, including endogenous H_2_O_2_ levels (A), mRNA expression of the catalase genes *ctl-1* (B), *ctl-2* (C), and *ctl-3* (D), and catalase activity (E). (F) H_2_O_2_ levels on adult day 2 after RNAi knockdown of specific *acox* genes, compared with the empty vector (EV) group. (G–J) Endogenous H_2_O_2_ levels (G, I) and Kaplan–Meier survival curves (H, J) in worms subjected to the indicated EGCG treatment combined with RNAi conditions (G, H) or N-acetylcysteine (NAC) co-treatment conditions (I, J). Survival assays were initiated at the young adult stage, defined as day 0 of adulthood. (A–G, I) Data are presented as mean ± SD from three biological replicates per group. (A–F) *P* values were calculated using an unpaired two-tailed t-test. (G, I) Statistical comparisons were performed using one-way ANOVA followed by Tukey’s multiple-comparison test. (H, J) *P* values were calculated using the log-rank test. Detailed lifespan data are provided in Table S6. CTL, control; EGCG, epigallocatechin gallate; NAC, N-acetylcysteine; EV, empty vector; NS, not significant; **P* < 0.05; ***P* < 0.01; ****P* < 0.001.

**Figure S10. AAK-2/AMPK, SKN-1/NRF2, and mitochondrial function contribute to EGCG-associated lifespan extension.** (A–E) Kaplan–Meier survival curves of (A) *eat-2(ad1116)*, (B) *bec-1* RNAi-treated, (C) *aak-2(ok524)*, (D) *skn-1(zu135)*, and (E) *isp-1(qm150)* worms treated with or without EGCG. Survival assays were initiated at the young adult stage, defined as day 0 of adulthood. Statistical significance was assessed by the log-rank test. Detailed lifespan data are provided in Table S6.

**Figure S11. Gene Ontology (GO) and KEGG pathway enrichment results of EGCG-associated human genes retrieved from the Comparative Toxicogenomics Database.** (A) Significantly enriched KEGG pathways. (B) Significantly enriched GO terms. All terms shown met the significance threshold of a corrected *P* value < 0.01. KEGG, Kyoto Encyclopedia of Genes and Genomes.

**Figure S12. Flowchart of participant selection in the National Health and Nutrition Examination Survey (NHANES).**

**Figure S13. Transcriptomic sampling scheme in *Caenorhabditis elegans.*** Baseline samples were collected at the onset of adulthood (adult day 0) before treatment. Worms were randomized to vehicle control (CTL; water) or EGCG (200 μM). Whole-worm samples were collected after continuous exposure at adult day 2 and adult day 8 for RNA extraction and RNA sequencing. Day designations indicated days since the onset of adulthood. N = 3 biological replicates per time point per condition. EGCG, epigallocatechin gallate. **Table S1. Top 20 U.S. Department of Agriculture (USDA) food codes ranked by contribution to total estimated dietary EGCG intake.**

| Main food description | Contribution (%) |
| --- | --- |
| Tea, hot, leaf, black | 27.13 |
| Tea, leaf, presweetened with sugar | 12.05 |
| Tea, hot, leaf, green | 9.94 |
| Tea, NS as to type, presweetened with sugar | 7.50 |
| Tea, NS as to type, unsweetened | 6.93 |
| Tea, NS as to type, presweetened, NS as to sweetener | 5.06 |
| Tea, NS as to type, presweetened with low calorie sweetener | 4.39 |
| Tea, iced, brewed, green, unsweetened | 3.79 |
| Tea, hot, leaf, black, decaffeinated | 2.85 |
| Tea, iced, brewed, black, pre-sweetened with sugar | 2.76 |
| Tea, iced, brewed, black, unsweetened | 2.27 |
| Tea, iced, instant, black, pre-sweetened with sugar | 1.93 |
| Tea, leaf, presweetened with low calorie sweetener | 1.62 |
| Tea, iced, instant, black, unsweetened | 1.54 |
| Tea, iced, instant, black, pre-sweetened with low calorie sweetener | 1.40 |
| Tea, iced, brewed, green, pre-sweetened with sugar | 1.07 |
| Tea, hot, leaf, green, decaffeinated | 1.05 |
| Tea, leaf, decaffeinated, presweetened with sugar | 0.87 |
| Tea, iced, brewed, green, decaffeinated, unsweetened | 0.77 |
| Tea, iced, brewed, green, pre-sweetened with low calorie sweetener | 0.69 |

**Table S2. Weighted multiple linear regression for the association of dietary EGCG intake categories with PhenoAge acceleration after excluding participants with extreme energy intake (N = 10,428).**

| **Variables** | **Model 1**  **β (95% CI)** | ***P* value** | **Model 2**  **β (95% CI)** | ***P* value** | **Model 3**  **β (95% CI)** | ***P* value** |
| --- | --- | --- | --- | --- | --- | --- |
| Categories of EGCG intake (mg/day) | | | | | | |
| Zero (0) | Reference | - | Reference | - | Reference | - |
| Low (≤ 1.52) | -0.88 (-1.34, -0.42) | **< 0.001** | -0.74 (-1.18, -0.30) | **0.002** | -0.57 (-1.00, -0.14) | **0.011** |
| High (> 1.52) | -1.06 (-1.55, -0.57) | **< 0.001** | -0.76 (-1.25, -0.28) | **0.003** | -0.63 (-1.07, -0.20) | **0.006** |

Note: Participants with extreme dietary energy intakes were excluded if their reported energy intake was below the 2.5% or above the 97.5% quantiles of the study sample. Model 1 was adjusted for age, sex, race, and poverty-to-income ratio. Model 2 was adjusted for Model 1 covariates plus smoking status, alcohol consumption, physical activity, and dietary energy intake. Model 3 was adjusted for Model 2 covariates plus hypertension and diabetes. EGCG, epigallocatechin gallate.

**Table S3. Survey and propensity score-weighted multiple linear regression for the association of dietary EGCG intake categories with PhenoAge acceleration in the overall population (N = 10,977).**

| **Variables** | **Model 1**  **β (95% CI)** | ***P* value** | **Model 2**  **β (95% CI)** | ***P* value** | **Model 3**  **β (95% CI)** | ***P* value** |
| --- | --- | --- | --- | --- | --- | --- |
| Categories of EGCG intake (mg/day) | | | | | | |
| Zero (0) | Reference | - | Reference | - | Reference | - |
| Low (≤ 1.42) | -0.54 (-0.98, -0.09) | **0.020** | -0.52 (-0.95, -0.10) | **0.016** | -0.53 (-0.97, -0.08) | **0.022** |
| High (> 1.42) | -0.64 (-1.13, -0.15) | **0.012** | -0.65 (-1.15, -0.15) | **0.013** | -0.67 (-1.13, -0.22) | **0.005** |

Note: Model 1 was adjusted for age, sex, race, and poverty-to-income ratio. Model 2 was adjusted for Model 1 covariates plus smoking status, alcohol consumption, physical activity, and dietary energy intake. Model 3 was adjusted for Model 2 covariates plus hypertension and diabetes. EGCG, epigallocatechin gallate.

**Table S4. Weighted multiple linear regression for the association between log_10_-transformed dietary EGCG intake and PhenoAge acceleration in the overall population (N = 10,977).**

| **Model** | **β (95% CI)** | ***P* value** |
| --- | --- | --- |
| Model 1 | -0.18 (-0.28, -0.09) | **< 0.001** |
| Model 2 | -0.15 (-0.24, -0.06) | **0.002** |
| Model 3 | -0.11 (-0.21, -0.01) | **0.026** |

Note: EGCG intake was log_10_-transformed and analyzed as a continuous variable. Model 1 was adjusted for age, sex, race, and poverty-to-income ratio. Model 2 was adjusted for Model 1 covariates plus smoking status, alcohol consumption, physical activity, and dietary energy intake. Model 3 was adjusted for Model 2 covariates plus hypertension and diabetes. EGCG, epigallocatechin gallate.

**Table S5. Weighted multivariable Cox proportional hazards models for the association of dietary EGCG intake categories with all-cause mortality in the overall population (N =10,964).**

| **Variables** | **Deaths/Total** | **Model 1**  **HRs (95% CI)** | ***P* value** | **Model 2**  **HRs (95% CI)** | ***P* value** | **Model 3**  **HRs (95% CI)** | ***P* value** |
| --- | --- | --- | --- | --- | --- | --- | --- |
| Categories of EGCG intake (mg/day) | | | | | | | |
| Zero (0) | 472/3851 | Reference | - | Reference | - | Reference | - |
| Low (≤ 1.39) | 408/3557 | 0.73 (0.61, 0.87) | **< 0.001** | 0.83 (0.69, 0.99) | **0.040** | 0.83 (0.69, 0.99) | **0.041** |
| High (> 1.39) | 396/3556 | 0.72 (0.60, 0.87) | **< 0.001** | 0.82 (0.68, 0.99) | **0.037** | 0.82 (0.68, 0.99) | **0.040** |

Note: Model 1 was adjusted for age, sex, race, and poverty-to-income ratio. Model 2 was adjusted for Model 1 covariates plus smoking status, alcohol consumption, physical activity, and dietary energy intake. Model 3 was adjusted for Model 2 covariates plus hypertension and diabetes. EGCG, epigallocatechin gallate; HR, hazard ratio.

**Table S6. Summary of lifespan experiments.**

| Data source | Strain | Bacteria/  RNAi | Chemical  Treatment | Number of worms | Mean lifespan  (days) ± SD | Comparison | Change in lifespan (%) | *P* value (log-rank) |
| --- | --- | --- | --- | --- | --- | --- | --- | --- |
| Figure 2A, B | N2 | OP50 | Vehicle | 117 | 17.05 ± 3.44 | - | - | - |
|  |  |  | 50 μM EGCG | 116 | 17.64 ± 3.90 | Vehicle | 3.46 | 0.201 |
|  |  |  | 100 μM EGCG | 119 | 18.03 ± 4.05 | Vehicle | 5.75 | 0.027 |
|  |  |  | 200 μM EGCG | 118 | 18.88 ± 4.04 | Vehicle | 10.73 | < 0.001 |
|  |  |  | 300 μM EGCG | 119 | 18.07 ± 3.52 | Vehicle | 5.98 | 0.039 |
| Figure S5 | N2 | heat-killed OP50 | Vehicle | 119 | 22.06 ± 4.43 | - | - | - |
|  |  |  | 200 μM EGCG | 117 | 24.66 ± 4.45 | Vehicle | 11.79 | < 0.001 |
| Figure 4F | N2 | HT115 | Vehicle | 117 | 16.96 ± 3.43 | - | - | - |
|  |  | HT115 | 200 μM EGCG | 116 | 18.92 ± 3.23 | HT115 + Vehicle | 11.56 | < 0.001 |
|  |  | *prx-5* RNAi | Vehicle | 118 | 16.69 ± 3.59 | - | - | - |
|  |  | *prx-5* RNAi | 200 μM EGCG | 116 | 16.83 ± 3.72 | *prx-5* RNAi + Vehicle | 0.84 | > 0.05 |
| Figure 5F | N2 | HT115 | Vehicle | 118 | 16.52 ± 4.20 | - | - | - |
|  |  | HT115 | 200 μM EGCG | 116 | 18.09 ± 4.49 | HT115 + Vehicle | 9.50 | < 0.01 |
|  |  | *dhs-28* RNAi | Vehicle | 119 | 16.75 ± 4.14 | - | - | - |
|  |  | *dhs-28* RNAi | 200 μM EGCG | 119 | 17.16 ± 4.61 | *dhs-28* RNAi + Vehicle | 2.45 | > 0.05 |

**Table S6. Summary of lifespan experiments (continued).**

| Data source | Strain | Bacteria/  RNAi | Chemical  Treatment | Number of worms | Mean lifespan  (days) ± SD | Comparison | Change in lifespan (%) | *P* value (log-rank) |
| --- | --- | --- | --- | --- | --- | --- | --- | --- |
| Figure S9H | N2 | HT115 | Vehicle | 116 | 16.91 ± 4.61 | - | - | - |
|  |  | HT115 | 200 μM EGCG | 118 | 19.06 ± 4.60 | HT115 + Vehicle | 12.71 | < 0.01 |
|  |  | *acox-1.2* RNAi | Vehicle | 115 | 16.65 ± 4.19 | - | - | - |
|  |  | *acox-1.2* RNAi | 200 μM EGCG | 117 | 17.27 ± 4.24 | *acox-1.2* RNAi + Vehicle | 3.72 | > 0.05 |
| Figure S9J | N2 | OP50 | Vehicle | 116 | 17.68 ± 4.49 | - | - | - |
|  |  |  | 200 μM EGCG | 115 | 19.71 ± 5.14 | Vehicle | 11.48 | < 0.01 |
|  |  |  | 5 mM NAC | 115 | 16.97 ± 4.30 | - | - | - |
|  |  |  | 200 μM EGCG+  5 mM NAC | 117 | 17.51 ± 4.68 | 5 mM NAC | 3.18 | > 0.05 |

**Table S6. Summary of lifespan experiments (continued).**

| Data source | Strain | Bacteria/  RNAi | Chemical  Treatment | Number of worms | Mean lifespan  (days) ± SD | Comparison | Change in lifespan (%) | *P* value (log-rank) |
| --- | --- | --- | --- | --- | --- | --- | --- | --- |
| Figure S10A | *eat-2(ad1116)* | OP50 | Vehicle | 111 | 20.87 ± 4.18 | - | - | - |
|  |  |  | 200 μM EGCG | 113 | 23.47 ± 4.30 | Vehicle | 12.46 | < 0.001 |
| Figure S10B | N2 | *bec-1* RNAi | Vehicle | 108 | 16.02 ± 3.12 | - | - | - |
|  |  | *bec-1* RNAi | 200 μM EGCG | 107 | 17.44 ± 2.67 | Vehicle | 8.86 | 0.003 |
| Figure S10C | *aak-2(ok524)* | OP50 | Vehicle | 107 | 13.86 ± 2.76 | - | - | - |
|  |  |  | 200 μM EGCG | 109 | 14.46 ± 2.68 | Vehicle | 4.33 | 0.105 |
| Figure S10D | *skn-1(zu135)* | OP50 | Vehicle | 104 | 13.86 ± 3.17 | - | - | - |
|  |  |  | 200 μM EGCG | 106 | 14.47 ± 3.28 | Vehicle | 4.4 | 0.152 |
| Figure S10E | *isp-1(qm150)* | OP50 | Vehicle | 110 | 23.67 ± 4.48 | - | - | - |
|  |  |  | 200 μM EGCG | 107 | 24.81 ± 4.68 | Vehicle | 4.82 | 0.071 |

**Table S7. *C. elegans* strains used in this study.**

| Strains | Strain name | Source | Reporter type and construct description |
| --- | --- | --- | --- |
| Wild type Bristol isolate | N2 | Caenorhabditis Genetics Center (CGC) | / |
| *dpy-5(e907)* I; *sEx*14661 [*rCes acox-1.2*::GFP + pCeh361] | BC14661 | CGC | Extrachromosomal GFP transcriptional reporter; GFP is expressed under regulatory sequences of *F08A8.2/acox-1.2* |
| *hjIs*37 [*vha-6p*::mRFP-PTS1 + Cbr*-unc-119(+)*] | VS10 | CGC | Integrated intestine-specific peroxisome reporter; *vha-6* promoter-driven mRFP fused to PTS1 labels intestinal peroxisomes |
| *sybIs*10775 [P*prx-19*-*prx-19* gDNA-mNeonGreen-*prx-19* 3′ UTR] | PHX10775 | SunyBiotech | Multi-copy integrated translational reporter comprising a 2,053-bp *prx-19* promoter, *prx-19* gDNA fused to mNeonGreen, and a 500-bp *prx-19* 3′ UTR |
| *sybIs*10751 [P*acox-3*-*acox-3* gDNA-mNeonGreen-*acox-3* 3′ UTR] | PHX10751 | SunyBiotech | Multi-copy integrated translational reporter comprising a 2,006-bp *acox-3* promoter, *acox-3* gDNA fused to mNeonGreen, and a 1,052-bp *acox-3* 3′ UTR |

**Table S7. *C. elegans* strains used in this study (continued).**

| Strains | Strain name | Source | Reporter type and construct description |
| --- | --- | --- | --- |
| *sybIs*10899 [P*prx-5*-*prx-5* gDNA-mNeonGreen-*prx-5* 3′ UTR, P*myo-2*::mCherry, pSL1190] | PHX10899 | SunyBiotech | Multi-copy integrated translational reporter comprising a 1,041-bp *prx-5* promoter, *prx-5* gDNA fused to mNeonGreen, and a 697-bp *prx-5* 3′ UTR; P*myo-2*::mCherry was used as a co-injection marker |
| *eat-2(ad1116)* II | DA1116 | CGC | / |
| *isp-1(qm150)* IV | MQ887 | CGC | / |
| *aak-2(ok524)* X | RB754 | CGC | / |
| *skn-1(zu135)* IV/nT1 [*unc-?(n754) let-?*] (IV;V) | EU31 | CGC | / |

**Table S8. Information on RNAi feeding clones obtained from the Vidal library and used in this study.**

| Target gene | WormBase ORF ID | RNAi clone ID | Vector | Bacterial host | Antibiotic selection | Annotated insert size (bp) | Chromosome | Genomic coordinates (WS112) |
| --- | --- | --- | --- | --- | --- | --- | --- | --- |
| *acox-1.6* | F59F4.1 | GHR-11082@H06 | pL4440-DEST | HT115(DE3) | Ampicillin, 100 μg/mL | 2004 | X | 15,838,464–15,833,741 |
| *acox-3* | F58F9.7 | GHR-11051@D03 | pL4440-DEST | HT115(DE3) | Ampicillin, 100 μg/mL | 2004 | IV | 6,248,484–6,245,331 |
| *prx-5* | C34C6.6 | GHR-11001@B01 | pL4440-DEST | HT115(DE3) | Ampicillin, 100 μg/mL | 1509 | II | 8,704,741–8,706,639 |
| *dhs-28* | M03A8.1 | GHR-10013@A04 | pL4440-DEST | HT115(DE3) | Ampicillin, 100 μg/mL | 1311 | X | 6,818,389–6,820,015 |
| *bec-1* | T19E7.3 | GHR-10039@F05 | pL4440-DEST | HT115(DE3) | Ampicillin, 100 μg/mL | 1128 | IV | 5,664,423–5,661,195 |

**Table S9. siRNA sequences used in AML12 cells.**

| Primer | 5'–3' Forward sequence | 5'–3' Reverse sequence |
| --- | --- | --- |
| *Pex5* | GGAUGUAGCUCAAGAUUAUAATT | UUAUAAUCUUGAGCUACAUCCTT |
| *Acox1* | GCAGCCAGAUUGGUAGAAAUUTT | AAUUUCUACCAAUCUGGCUGCTT |
| NC | UUCUCCGAACGUGUCACGUTT | ACGUGACACGUUCGGAGAATT |

**Table S10. Primers used for quantitative RT–PCR.**

| Primer | 5'–3' Forward sequence | 5'–3' Reverse sequence |
| --- | --- | --- |
| *act-1* | CTTGCCCCATCAACCATGA | CAAGTTGACGAAGTTGTGCAT |
| *prx-1* | GGCAATGGAGAAAGTAGTATCAGT | CACAACGGTCTTCCTTAGTCAAT |
| *prx-2* | TGCCTCTATGGGTTGCCAGA | TCCCGCGTATGCAATATCCA |
| *prx-3* | AGCTGCACTGAAGATGGTAGA | ACTGGTGAATATCGGAGGAATACA |
| *prx-5* | TTACCACAGGCATCAGCATC | TGTTGACTCCATTGACTCGC |
| *prx-6* | CGAAGTTGATGGATATGAGATGGT | CTGTAAGATACGGCGGTCTAATG |
| *prx-11* | CGCAGGAGGATATTTTGACGC | GCATCGACAACTTTCCAGCC |
| *prx-12* | CTGGAATCGTTTCAAGGCTCT | CAAATTGGGCATTTATTGGTGT |
| *prx-13* | CGCTCCACATTATACTGCTCAA | GACTTCCATCGGCAACACTT |
| *prx-14* | CTCAGTTGCTAACAGAGATGCT | AGAAGTTGTAGTTGTGGTGATTGA |
| *prx-19* | ACCAGCACCAAAACCACGTA | AGTGGCTGTTCTCCTTCTCC |
| *acox-1.1* | AAGACAGATGTTCTTCCGTC | ACTATTTATAAGTGCAATGTTTG |
| *acox-1.2* | CTTCTTTTTTGTTGATATCAGTAAC | GCTGCCATCTTATCCGTATC |
| *acox-1.3* | TGATGTTCTACCGTCAGTCG | TACCAGGGCGGGTGGAAGTT |
| *acox-1.4* | GGGATGGTAATGTCTACGAG | CCAGATGAGAACAGAAACTT |
| *acox-1.5* | CAGTACTCGGTCGCCGTGAT | TACACCGCACGAATCGAAAG |
| *acox-1.6* | CTGAAATTTTTGAAATGAGTCG | GTCATTGCCACAACTTTACG |
| *acox-3* | AACAACCAAGATGGGTTTGC | CTGGATTTATTGGGAAATATAC |
| *daf-22* | CATCCGTGTTCGCTGAGAAT | GACCTGGACATCATTTGGAGTAAG |
| *dhs-28* | GGAAAACTGAAAGTGAAGGG | CAATAATTAATCATTCTGACTCA |
| *maoc-1* | AAGTGCTTCCATCGTACATTGTT | CCTTCTGCTGAAAGTGGTTGAT |
| *ctl-1* | GTGTCGTTCATGCCAAGGGA | GATTCTCCAGCGACCGTTGA |
| *ctl-2* | CTACAGTCGGTGGTGAGAGC | TCGGGAAGTGGATAGGGTCA |
| *ctl-3* | GTGTCGTCCATGCCAAAGGT | GATCCCGATTCTCCAGCGAC |

**Table S11. Antibodies used for western blot analysis.**

| Antibody | Company | Catalog number | Dilution |
| --- | --- | --- | --- |
| p21 | Immunoway | YM8364 | 1:5000 |
| p16 | Immunoway | YM8152 | 1:1000 |
| PEX5 | Cell Signaling Technology | #83020 | 1:1000 |
| PEX19 | Proteintech | 14713-1-AP | 1:2000 |
| ABCD1 | Proteintech | 83038-4-RR | 1:10000 |
| PMP70 | Cell Signaling Technology | #94560 | 1:1000 |
| ACOX1 | Proteintech | 10957-1-AP | 1:4000 |
| EHHADH | Proteintech | 26570-1-AP | 1:3000 |
| HSD17B4 | Boster | A02923 | 1:1000 |
| ACAA1 | Proteintech | 12319-2-AP | 1:1000 |
| β-actin | Abways | AB0035 | 1:10000 |
| α-tubulin | Proteintech | 66031-1-Ig | 1:10000 |
| HRP-Goat Anti-Rabbit IgG Conformation Specific Recombinant Secondary Antibody | Proteintech | RGAR301 | 1:10000 |
| HRP-conjugated Goat Anti-Mouse IgG(H+L) | Proteintech | SA00001-1 | 1:10000 |

**Supplementary Methods**

**Survey weighting**

The NHANES survey design was specified using SDMVSTRA as the stratification variable and SDMVPSU as the primary sampling unit variable. Because EGCG intake was derived from two-day dietary recall data, the Day 2 dietary recall sample weight (WTDR2D) was applied. As three 2-year NHANES cycles were combined, a 6-year dietary sample weight was constructed by dividing WTDR2D by 3, in accordance with the NHANES analytic guidelines. These survey design specifications and weights were applied to all weighted regression analyses.

**Assessment of dietary catechin intake**

Dietary catechin intakes were obtained from the U.S. Department of Agriculture (USDA) Flavonoid Intake Data Files for What We Eat in America (WWEIA), NHANES, covering the 2007–2008, 2009–2010, and 2017–2018 cycles. USDA estimated daily flavonoid intakes by linking foods and beverages reported in the 24-h dietary recalls to flavonoid composition values in the Database of Flavonoid Values for USDA Food Codes using food codes. For each respondent, the amount of each item consumed was multiplied by its corresponding flavonoid concentration, and the resulting intakes were summed across all reported items. Respondent-level daily intakes of EGCG, epicatechin, epigallocatechin, epicatechin gallate, catechin, and gallocatechin were linked to NHANES participant data using the unique respondent identifier (SEQN). For participants with valid recalls on both days, intake of each catechin was expressed as the mean of the two daily estimates (mg/day). Food-level linkage, assignment of flavonoid composition values, and calculation of daily flavonoid intakes were performed by USDA.

**Dietary source attribution of EGCG intake**

To characterize the major dietary sources of EGCG intake, food-level dietary recall records from the 2007–2008, 2009–2010, and 2017–2018 NHANES cycles were linked to the corresponding USDA Flavonoid Intake Data Files. Analyses were restricted to participants included in the analytic sample. Estimated EGCG amounts from Day 1 and Day 2 dietary recalls were aggregated by USDA food code across the three NHANES periods. Food codes were then linked to their corresponding main food descriptions using the USDA MainFoodDesc files for the 2007–2010 and 2017–2018 periods. The contribution of each food code was calculated as its aggregated estimated EGCG amount relative to total estimated EGCG intake across all included dietary records. Food codes with main food descriptions containing “tea” were categorized as tea-based foods and beverages, whereas all remaining food codes were classified as non-tea foods and beverages. Tea-based food codes were further categorized as black tea, green tea, other specified tea, including oolong tea and chai, or tea of unspecified type, according to the corresponding main food descriptions.

**Partial least squares (PLS) regression**

PLS regression was performed in R using the pls package to assess the relative contributions of the six individual catechins to phenotypic age acceleration. Model performance was evaluated using 10-fold cross-validation. The optimal number of latent components was selected by comparing the cross-validated predictive ability index (Q^2^) and the root mean squared error of cross-validation (RMSECV) across candidate models. The model with the highest cross-validated Q^2^ and the lowest RMSECV was selected for subsequent analyses. Variable importance in projection (VIP) scores were then calculated from the selected model to quantify and rank the relative contribution of each individual catechin to the prediction of PhenoAge acceleration. Higher VIP scores indicate greater relative contribution to the PLS model.

**Random forest regression**

Random forest regression was performed using the ranger package in R, with PhenoAge acceleration as the outcome and the six catechins as predictors. The model included 500 trees, with two candidate predictors considered at each split. Predictor importance was assessed using permutation importance based on out-of-bag prediction error. For each catechin, its values were permuted in the corresponding out-of-bag observations, and the resulting increase in mean squared error was expressed as a percentage of the overall forest out-of-bag mean squared error (%IncMSE). Larger %IncMSE values indicate greater relative predictive importance.

**RNA sequencing and bioinformatic analysis**

Total RNA was isolated using TRIzol. Sequencing libraries were constructed and sequenced on an Illumina NovaSeq X Plus system by Genedenovo Biotechnology (Guangzhou, China). RNA-sequencing data processing and bioinformatic analyses were conducted using the Genedenovo online analysis platform and R version 4.4.3. DEGs were identified using DESeq2, defined by an FDR < 0.05 and |log₂(fold change)| > 1. GO and KEGG functional enrichments were performed with multiple-testing correction (Q-value < 0.05). GSEA significance was defined as |NES| > 1, nominal *P* < 0.05, and FDR *q* < 0.25. Short Time-series Expression Miner (STEM) identified expression patterns relative to day 0. Two three-point series were analyzed: (i) day 0, control day 2, and EGCG day 2; and (ii) day 0, control day 8, and EGCG day 8. Input genes comprised the union of age-associated DEGs (control vs baseline) and EGCG-associated DEGs (EGCG vs control at the same time point). Significantly enriched profiles (*P* < 0.05) were selected. PPI networks of high-confidence interactions (combined score ≥ 0.70) were constructed using STRING, evaluating network topology and hub genes via the R package igraph.

**Widely targeted quantitative lipidomic analysis**

Lipids were extracted using methyl tert-butyl ether/methanol (3:1, v/v) with internal standards, followed by water-induced phase separation. The dried organic phase was reconstituted in acetonitrile/isopropanol (1:1, v/v). Lipid profiling was performed using ultra-performance liquid chromatography–tandem mass spectrometry (ExionLC™ AD & QTRAP® 6500+ System; SCIEX) with a Thermo Accucore™ C30 column. Lipids were identified via the MetWare database and quantified in multiple reaction monitoring mode. Concentrations were calculated using internal standards and normalized to total protein. Lipidomic data processing and statistical analyses were conducted using the MetWare online analysis platform. Differential lipids were defined by VIP > 1 from orthogonal partial least squares discriminant analysis and *P* < 0.05 (two-sided Student’s t-test). Assays were performed by Metware Biotechnology (Wuhan, China).

**Hydrogen peroxide assay**

Hydrogen peroxide levels were measured using the Amplex® Red Hydrogen Peroxide/Peroxidase Assay Kit (Thermo Fisher Scientific). Worms were collected and washed three times with M9 buffer (22 mM KH_2_PO_4_, 42 mM Na_2_HPO4, 86 mM NaCl, and 1 mM MgSO4) to remove residual *Escherichia coli* OP50. Worm pellets were resuspended in 500 μL of the 1× reaction buffer supplied with the kit and mixed with 500 μL of freshly prepared Amplex Red/HRP working solution prepared according to the manufacturer’s instructions. Samples were incubated for 1 h at room temperature in the dark and then centrifuged at 800 × g for 3 min. Supernatants (100 μL) were transferred to a 96-well plate, and fluorescence was measured at excitation and emission wavelengths of 540 and 590 nm, respectively. Hydrogen peroxide levels were normalized to the total protein content. Three independent biological replicates were analyzed per group.

**Catalase activity assay**

Catalase (CAT) activity was measured using a commercially available ammonium molybdate colorimetric assay kit (Boxbio, Beijing, China) according to the manufacturer’s instructions. Worm samples were homogenized on ice in extraction buffer and centrifuged at 8,000 × g for 10 min at 4 ℃. The resulting supernatants were collected as crude enzyme extracts. The assay reactions and corresponding sample controls were prepared, and absorbance was measured at 405 nm using a microplate reader. Total protein concentration was determined using a bicinchoninic acid (BCA) protein assay kit. CAT activity was normalized to the total protein content.

**Bicinchoninic acid (BCA) protein assay**

Protein concentrations were determined using the Pierce BCA Protein Assay Kit (Thermo Fisher Scientific, USA). Briefly, the working reagent was freshly prepared by mixing Reagent A and Reagent B at a ratio of 50:1. For the microplate assay, 25 μL of each BSA standard or appropriately diluted sample was added to a 96-well microplate, followed by 200 μL of working reagent. After thorough mixing, the plate was incubated at 37°C for 30 min. The plate was then cooled to room temperature, and absorbance was measured at 562 nm using a microplate reader.

**EGFP–SKL plasmid construction**

The EGFP–SKL expression plasmid was constructed by Hanbio Biotechnology Co., Ltd. (Shanghai, China). The type 1 peroxisomal targeting signal Ser-Lys-Leu (SKL) was fused in frame to the C terminus of EGFP in the pEGFP vector. The resulting pEGFP–SKL construct was verified by Sanger sequencing.

**Generation of multi-copy integrated fluorescent reporter strains**
Multi-copy integrated fluorescent reporter strains PHX10751, PHX10899, and PHX10775 were generated by SunyBiotech (Fuzhou, China). Briefly, a 10-μL DNA injection mixture containing the respective target plasmid and reporter marker plasmid was microinjected into the gonads of wild-type *Caenorhabditis elegans* hermaphrodites. Approximately 30 injected P0 hermaphrodites were cultured, and transgenic F1 progeny were individually transferred to separate nematode growth medium plates. Following progeny production, extrachromosomal transgenic lines with relatively stable inheritance were identified and maintained. Approximately 200 L4-stage animals from each selected extrachromosomal line were subjected to X-ray irradiation. Following recovery, irradiated animals were individually plated and screened, and lines exhibiting 100% germline transmission of the transgene were retained as fully integrated multicopy transgenic strains.

**Construction of RNAi feeding plasmids**

Custom RNAi feeding plasmids targeting *C. elegans acox-1.1*, *acox-1.2*, *acox-1.3*, *acox-1.4*, and *acox-1.5* were generated by SunyBiotech. The corresponding cDNA inserts were synthesized and assembled into the XbaI/KpnI-linearized L4440 vector by Gibson assembly. The resulting plasmids were transformed into *Escherichia coli* HT115(DE3) and selected on LB agar containing ampicillin (100 μg/mL). Individual positive colonies were expanded in LB broth supplemented with ampicillin, screened by PCR using amplification primers, and confirmed by DNA sequencing.

**Sequence S1. L4440-*acox-1.1* cDNA**

AACCTGGCTTATCGAAATTAATACGACTCACTATAGGGAGACCGGCAGATCTGATATCATCGATGAATTCGAGCTCCACCGCGGTGGCGGCCGCTCTAGAATGGTGCATCTCAACAAAACAATTCAGGAGGGTGATAACCCGGATCTCACTGCCGAACGCTTGACGGCAACATTTGATACTCATGCGATGGCTGCACAGATTTATGGGGGAGAAATGCGCGCCCGTCGTCGTCGTGAGATCACCGCGAAGCTTGCAGAAATCCCAGAGCTCCATGACTCGATGCCACTTCCGTATATGACAAGAGAGGAGAAAATTATGGAAAGTGCTAGGAAACTGACGGTTTTGACACAAAGAATGTCAGAGATCATTGATCCAACTGATGCAGGCGAGTTGTATCATTTGAACAACGAAGTTCTCGGAATCGAAGGAAATCCTATGGCTCTTCACGGTGTGATGTTCATTCCAGCCCTCAACGCTCAAGCTAGTGATGAGCAGCAAGCCAAGTGGCTCATTCGAGCGCTCCGACGAGAAATCATCGGAACCTACGCCCAAACTGAAATGGGTCATGGCACAAATCTTCAAAATCTGGAAACAACTGCTACTTATGACATTGGAACTCAGGAGTTTGTGCTTCACACTCCAAAGATCACCGCATTGAAGTGGTGGCCAGGGAATCTTGGAAAAAGCAGCAACTATGCAGTTGTAGTTGCACATATGTATATAAAGGGAAAGAACTTTGGACCACATACCTTCATGGTTCCATTGAGAGACGAGAAAACTCACAAACCACTTCCAGGGATTACAATTGGAGATATTGGACCAAAAATGGCTTATAATATTGTAGACAATGGATTCCTAGGCTTCAACAACTACCGTATCCCAAGAACGAATCTTTTGATGAGACATACCAAAGTAGAGGCTGACGGGACTTATATCAAACCACCACATGCAAAGATCAATTACTCTGCAATGGTACATGTGAGAAGTTATATGCTTACTGGACAGGCGATTATGTTGTCGTATGCGTTGAATATTGCAACAAGATACTCGGCAGTGAGAAGACAGGGGCAGATTGATAAAAATGAACCAGAGGTAAAAGTGCTCGAGTACCAAACCCAGCAGCACCGTCTCTTCCCATTCATCGCCCGTGCCTACGCTTTCCAGTTTGCTGGTGCCGAGACCGTGAAGCTTTACGAACGAGTACTCAAGGAGATGAAATCTGGAAATGTGTCTCTTATGGCTGACTTACATGCATTGACTTCTGGTTTAAAATCAGTTGTTACTCATCAAACTGGAGAAGGTATCGAACAAGCGAGAATGGCTTGTGGTGGGCATGGATATTCTATGGCTAGTTACATCTCTGAAATCTACGGAGTTGCAATTGGAGGATGCACGTATGAAGGAGAAAATATGGTTATGCTTCTTCAACTCGCTCGTTACCTCGTCAAATCCGCTGCCCTTGTGAAGTCCGGAAAAGCTTCTCAGCTTGGACCACTGGTAGCCTACTTGGGCGCCAGAAGTGAGCCAACTAGCCTGATAGATAGAGTTCCAAATGGTGGAATCACGGAATACATCAAGACATTCCAACATATCGCAAAGAGACAGACGTTGAAGGCTGCCAACAAGTTCTTCGGATTAATGGAGAATGGAGAAAAGAGAGAAATCGCCTGGAACAAGTCATCCGTGGAGTTGAATAGGGCGAGTCGCCTCCACACCCGCCTCTTCATCGTCGAAGCCTTTGCCAGACGAGTCAACGAAATCGGAGATATCACCATCAAAGAGGCTCTCAGCGATCTCCTCCATCTTCACGTCAACTATGAACTTTTGGATGTTGCCACGTATGCACTGGAAGATGGATTCATGTCATCCACTCAGTTGGATTATGTCAGAGATCAATTGTACTTCTATCTCCAGAAAATTCGACCAAACGCAGTTTCATTGCTAGATTCCTGGGAGTTTAGTGATAGAGAGCTGAGATCTGTCCTAGGCCGCCGTGATGGTCATGTCTACGAGAACCTTTTCAAGTGGGCAAAGGAAAGTCCGTTGAACAAGACAGATGTTCTTCCGTCAGTGGATACATATTTGAAGCCAATGATGGAAAAAGCCAGGCAGAGCAAGCTATAAGGTACCCAATTCGCCCTATAGTGAGTCGTATTACGCGCGCTCACTGGCCGTCGTTTTACAACGTCGTGACTGGGAAAACCCTGGCGTTACCCAACTTAATCGCCTTGCAGCACATCCCCCTTTCGCCAGCTGGCGTAATAGCGAAGAGGCCCGCACCGATCGCCCTTCCCAACAGTTGCGCAGCCTGAATGGCGAATGGGACGCGCCCTGTAGCGGCGCATTAAGCGCGGCGGGTGTGGTGGTTACGCGCAGCGTGACCGCTACACTTGCCAGCGCCCTAGCGCCCGCTCCTTTCGCTTTCTTCCCTTCCTTTCTCGCCACGTTCGCCGGCTTTCCCCGTCAAGCTCTAAATCGGGGGCTCCCTTTAGGGTTCCGATTTAGTGCTTTACGGCACCTCGACCCCAAAAAACTTGATTAGGGTGATGGTTCACGTAGTGGGCCATCGCCCTGATAGACGGTTTTTCGCCCTTTGACGTTGGAGTCCACGTTCTTTAATAGTGGACTCTTGTTCCAAACTGGAACAACACTCAACCCTATCTCGGTCTATTCTTTTGATTTATAAGGGATTTTGCCGATTTCGGCCTATTGGTTAAAAAATGAGCTGATTTAACAAAAATTTAACGCGAATTTTAACAAAATATTAACGCTTACAATTTAGGTGGCACTTTTCGGGGAAATGTGCGCGGAACCCCTATTTGTTTATTTTTCTAAATACATTCAAATATGTATCCGCTCATGAGACAATAACCCTGATAAATGCTTCAATAATATTGAAAAAGGAAGAGTATGAGTATTCAACATTTCCGTGTCGCCCTTATTCCCTTTTTTGCGGCATTTTGCCTTCCTGTTTTTGCTCACCCAGAAACGCTGGTGAAAGTAAAAGATGCTGAAGATCAGTTGGGTGCACGAGTGGGTTACATCGAACTGGATCTCAACAGCGGTAAGATCCTTGAGAGTTTTCGCCCCGAAGAACGTTTTCCAATGATGAGCACTTTTAAAGTTCTGCTATGTGGCGCGGTATTATCCCGTATTGACGCCGGGCAAGAGCAACTCGGTCGCCGCATACACTATTCTCAGAATGACTTGGTTGAGTACTCACCAGTCACAGAAAAGCATCTTACGGATGGCATGACAGTAAGAGAATTATGCAGTGCTGCCATAACCATGAGTGATAACACTGCGGCCAACTTACTTCTGACAACGATCGGAGGACCGAAGGAGCTAACCGCTTTTTTGCACAACATGGGGGATCATGTAACTCGCCTTGATCGTTGGGAACCGGAGCTGAATGAAGCCATACCAAACGACGAGCGTGACACCACGATGCCTGTAGCAATGGCAACAACGTTGCGCAAACTATTAACTGGCGAACTACTTACTCTAGCTTCCCGGCAACAATTAATAGACTGGATGGAGGCGGATAAAGTTGCAGGACCACTTCTGCGCTCGGCCCTTCCGGCTGGCTGGTTTATTGCTGATAAATCTGGAGCCGGTGAGCGTGGGTCTCGCGGTATCATTGCAGCACTGGGGCCAGATGGTAAGCCCTCCCGTATCGTAGTTATCTACACGACGGGGAGTCAGGCAACTATGGATGAACGAAATAGACAGATCGCTGAGATAGGTGCCTCACTGATTAAGCATTGGTAACTGTCAGACCAAGTTTACTCATATATACTTTAGATTGATTTAAAACTTCATTTTTAATTTAAAAGGATCTAGGTGAAGATCCTTTTTGATAATCTCATGACCAAAATCCCTTAACGTGAGTTTTCGTTCCACTGAGCGTCAGACCCCGTAGAAAAGATCAAAGGATCTTCTTGAGATCCTTTTTTTCTGCGCGTAATCTGCTGCTTGCAAACAAAAAAACCACCGCTACCAGCGGTGGTTTGTTTGCCGGATCAAGAGCTACCAACTCTTTTTCCGAAGGTAACTGGCTTCAGCAGAGCGCAGATACCAAATACTGTCCTTCTAGTGTAGCCGTAGTTAGGCCACCACTTCAAGAACTCTGTAGCACCGCCTACATACCTCGCTCTGCTAATCCTGTTACCAGTGGCTGCTGCCAGTGGCGATAAGTCGTGTCTTACCGGGTTGGACTCAAGACGATAGTTACCGGATAAGGCGCAGCGGTCGGGCTGAACGGGGGGTTCGTGCACACAGCCCAGCTTGGAGCGAACGACCTACACCGAACTGAGATACCTACAGCGTGAGCTATGAGAAAGCGCCACGCTTCCCGAAGGGAGAAAGGCGGACAGGTATCCGGTAAGCGGCAGGGTCGGAACAGGAGAGCGCACGAGGGAGCTTCCAGGGGGAAACGCCTGGTATCTTTATAGTCCTGTCGGGTTTCGCCACCTCTGACTTGAGCGTCGATTTTTGTGATGCTCGTCAGGGGGGCGGAGCCTATGGAAAAACGCCAGCAACGCGGCCTTTTTACGGTTCCTGGCCTTTTGCTGGCCTTTTGCTCACATGTTCTTTCCTGCGTTATCCCCTGATTCTGTGGATAACCGTATTACCGCCTTTGAGTGAGCTGATACCGCTCGCCGCAGCCGAACGACCGAGCGCAGCGAGTCAGTGAGCGAGGAAGC

**Sequence S2. L4440*-acox-1.2* cDNA**

AACCTGGCTTATCGAAATTAATACGACTCACTATAGGGAGACCGGCAGATCTGATATCATCGATGAATTCGAGCTCCACCGCGGTGGCGGCCGCTCTAGAATGGCGAACCGATCAATTCGTGATGGAGATAACCCCGAATTGCTGGAAGAACGGAGGATGGCAACTTTTGATACGGATAAGATGGCAGCGGTGATCTATGGAAGTGAAGAGTTCGCTCGTCGCCGTCGTGAGATCACCGACGCCGTGTCCAAGATCCCTGAGCTCGCCGACATCAAGCCCTATCCATTTTTGACTCGAGAGGAGAAGGTGACCGAGGGCACCAGAAAAATATCGATTTTGACAAAATATCTGAATCAGCTGATTGATAGAGATAATGAAGAAGAAAGTTTGCACCTGCATCGAGAAGTGATTGGCTACGAGGGTCATCCCTTCGCTCTCCATGACGCTTTGTTTATTCCAACCCTTCAGTCACAGGCTAGTGATGAACAGCAGGAGAAATGGCTCGAGAGAGCTCGGCGACGAGAGATCATTGGATGTTATGCGCAAACTGAGCTCGGTCACGGCTCAAATCTTAGGAATCTTGAAACAACTGCAGTCTATGACATTGCTTCCCAGGAGTTTGTACTACATACTCCTACGACAACGGCGTTGAAGTGGTGGCCTGGAGCACTTGGGAAGAGCTGTAACTATGCATTGGTTGTAGCTGAACTGATTATAAAGAGAAATAACTATGGCCCCCACTTTTTTATGGTGCAGCTGAGAGATGAAAAAACCCATATTCCACTAAAAGGCGTTACCGTAGGAGATATCGGGCCTAAAATGAACTTCAATGCGGCGGATAATGGGTACCTTGGGTTGAACAACTTGAGAGTTCCACGAACAAATCTTTTGATGAGACATTGCAAAGTAGAGGCGGATGGGACGTATGTGAAGCCACCGCATGCAAAGATCGGGTACTCTGGAATGGTGAAGATCAGAAGTCAGATGGCTATGGAGCAGGGGTTGTTTTTAGCACATGCATTGACAATTGCAGCGAGGTATTCGGCAGTGAGGAGGCAAGGACATTTGGATGATAAGCAAGTCGAAGTAAAAGTCCTTGACTACCAGACCCAGCAGCATCGTCTATTCCCATCATTAGCCCGTGCGTATGCATTTATATTCACTGGATTTGAGACAATTCACTTATATTCTCAATTATTAAAAGATGTTGATATGGGTAATACATCTGGAATGGCTGATTTACATGCATTGACTTCTGGTTTAAAATCAGTTGTTGCTCATGAAACTGGAGAAGGTATTGAACAGGCGAGGATGGCTTGTGGTGGACATGGATACTCTATGGCTAGTTATATATCTGTCGTATATGGGATTGCTATTGGCGGATGCACTTATGAAGGGGAGAACATGGTGATGCTCCTGCAACTTGCACGGTATTTAGTGAAATCCGTGGAACTTATCAAGGCCGGTAAAGCTAAGAAATTAGGACCAGTGGCATCATATCTAGCAGACAAGAGTGATGAAACTGATCTAACCAGCCTGAATGGATATGTAAAGATGTTCGAGAACATGGCTAGAAGGCAGGCTTGGAAAGCAACAGAGAAATTCTTGAAATTGATGGAAAGTGGGGAATCAAGAGAAGTGGCATGGAATAAATCAGCAGTGGAGTTGACTAGAGCTAGCCGTCTACACACCCGTCTATTCATAATCGAAGCTTTTATGAGAAGAGTGTCTCGAATTGAAGATATTCCAGTGAAAGAGGTTCTAACAGATCTTCTCCATCTTCATGTCAACTATGAACTATTGGATGTTGCCACGTATGCACTGGAATTCATGTCATTCACTCAGTTGGATTATGTTAGAGATCAACTATATTTGTATCTGGAGAAGATTCGACCAAATGCAGTATCTCTAGTTGACTCTTTTCAGATTAGTGATATGCAATTGAGATCTGTCCTTGGCCGCCGGGATGGTCACGTCTACGAGAATCTCTTCAAATGGGCAAAGTCAAGTCCCTTGAACAACGCAGATGTTCTACCGTCAGTCGAGAAGTACTTAAAGCCTATGATGGAAAAGGCGAAGCTTTAAGGTACCCAATTCGCCCTATAGTGAGTCGTATTACGCGCGCTCACTGGCCGTCGTTTTACAACGTCGTGACTGGGAAAACCCTGGCGTTACCCAACTTAATCGCCTTGCAGCACATCCCCCTTTCGCCAGCTGGCGTAATAGCGAAGAGGCCCGCACCGATCGCCCTTCCCAACAGTTGCGCAGCCTGAATGGCGAATGGGACGCGCCCTGTAGCGGCGCATTAAGCGCGGCGGGTGTGGTGGTTACGCGCAGCGTGACCGCTACACTTGCCAGCGCCCTAGCGCCCGCTCCTTTCGCTTTCTTCCCTTCCTTTCTCGCCACGTTCGCCGGCTTTCCCCGTCAAGCTCTAAATCGGGGGCTCCCTTTAGGGTTCCGATTTAGTGCTTTACGGCACCTCGACCCCAAAAAACTTGATTAGGGTGATGGTTCACGTAGTGGGCCATCGCCCTGATAGACGGTTTTTCGCCCTTTGACGTTGGAGTCCACGTTCTTTAATAGTGGACTCTTGTTCCAAACTGGAACAACACTCAACCCTATCTCGGTCTATTCTTTTGATTTATAAGGGATTTTGCCGATTTCGGCCTATTGGTTAAAAAATGAGCTGATTTAACAAAAATTTAACGCGAATTTTAACAAAATATTAACGCTTACAATTTAGGTGGCACTTTTCGGGGAAATGTGCGCGGAACCCCTATTTGTTTATTTTTCTAAATACATTCAAATATGTATCCGCTCATGAGACAATAACCCTGATAAATGCTTCAATAATATTGAAAAAGGAAGAGTATGAGTATTCAACATTTCCGTGTCGCCCTTATTCCCTTTTTTGCGGCATTTTGCCTTCCTGTTTTTGCTCACCCAGAAACGCTGGTGAAAGTAAAAGATGCTGAAGATCAGTTGGGTGCACGAGTGGGTTACATCGAACTGGATCTCAACAGCGGTAAGATCCTTGAGAGTTTTCGCCCCGAAGAACGTTTTCCAATGATGAGCACTTTTAAAGTTCTGCTATGTGGCGCGGTATTATCCCGTATTGACGCCGGGCAAGAGCAACTCGGTCGCCGCATACACTATTCTCAGAATGACTTGGTTGAGTACTCACCAGTCACAGAAAAGCATCTTACGGATGGCATGACAGTAAGAGAATTATGCAGTGCTGCCATAACCATGAGTGATAACACTGCGGCCAACTTACTTCTGACAACGATCGGAGGACCGAAGGAGCTAACCGCTTTTTTGCACAACATGGGGGATCATGTAACTCGCCTTGATCGTTGGGAACCGGAGCTGAATGAAGCCATACCAAACGACGAGCGTGACACCACGATGCCTGTAGCAATGGCAACAACGTTGCGCAAACTATTAACTGGCGAACTACTTACTCTAGCTTCCCGGCAACAATTAATAGACTGGATGGAGGCGGATAAAGTTGCAGGACCACTTCTGCGCTCGGCCCTTCCGGCTGGCTGGTTTATTGCTGATAAATCTGGAGCCGGTGAGCGTGGGTCTCGCGGTATCATTGCAGCACTGGGGCCAGATGGTAAGCCCTCCCGTATCGTAGTTATCTACACGACGGGGAGTCAGGCAACTATGGATGAACGAAATAGACAGATCGCTGAGATAGGTGCCTCACTGATTAAGCATTGGTAACTGTCAGACCAAGTTTACTCATATATACTTTAGATTGATTTAAAACTTCATTTTTAATTTAAAAGGATCTAGGTGAAGATCCTTTTTGATAATCTCATGACCAAAATCCCTTAACGTGAGTTTTCGTTCCACTGAGCGTCAGACCCCGTAGAAAAGATCAAAGGATCTTCTTGAGATCCTTTTTTTCTGCGCGTAATCTGCTGCTTGCAAACAAAAAAACCACCGCTACCAGCGGTGGTTTGTTTGCCGGATCAAGAGCTACCAACTCTTTTTCCGAAGGTAACTGGCTTCAGCAGAGCGCAGATACCAAATACTGTCCTTCTAGTGTAGCCGTAGTTAGGCCACCACTTCAAGAACTCTGTAGCACCGCCTACATACCTCGCTCTGCTAATCCTGTTACCAGTGGCTGCTGCCAGTGGCGATAAGTCGTGTCTTACCGGGTTGGACTCAAGACGATAGTTACCGGATAAGGCGCAGCGGTCGGGCTGAACGGGGGGTTCGTGCACACAGCCCAGCTTGGAGCGAACGACCTACACCGAACTGAGATACCTACAGCGTGAGCTATGAGAAAGCGCCACGCTTCCCGAAGGGAGAAAGGCGGACAGGTATCCGGTAAGCGGCAGGGTCGGAACAGGAGAGCGCACGAGGGAGCTTCCAGGGGGAAACGCCTGGTATCTTTATAGTCCTGTCGGGTTTCGCCACCTCTGACTTGAGCGTCGATTTTTGTGATGCTCGTCAGGGGGGCGGAGCCTATGGAAAAACGCCAGCAACGCGGCCTTTTTACGGTTCCTGGCCTTTTGCTGGCCTTTTGCTCACATGTTCTTTCCTGCGTTATCCCCTGATTCTGTGGATAACCGTATTACCGCCTTTGAGTGAGCTGATACCGCTCGCCGCAGCCGAACGACCGAGCGCAGCGAGTCAGTGAGCGAGGAAGC

**Sequence S3. L4440-*acox-1.3* cDNA**

AACCTGGCTTATCGAAATTAATACGACTCACTATAGGGAGACCGGCAGATCTGATATCATCGATGAATTCGAGCTCCACCGCGGTGGCGGCCGCTCTAGAATGAGCTCAATTTGCAAAGGAGATAACTCAGATCTCACAGAAGAACGAAAGAATGCGACTTTTGATACGGATAAGATGGCTGCCGTGATATATGGACGTGAAGAGATTGCTAGTCGCCGTCGACAACTCACTGAATCCATCTCCCGGATCCATGAGCTTGCAGAATCCAAACCCCTAGTTTTCATGACACGAGAGGAAAAAATCGCTGAGTCCTGTAGAAAGCTGGAGGTTTTGAGTCGACACTGGAATCAGACCCCGTTCAACAGAGACAACGAAGAAGATGCACTGCACATCTACAGAGAGGTATTGGGGATGGAGGGACATCCGTTGGCGCTTCACGATACAATGTTCATACCGACTTTGGTTGCACAGGCGAGCCAAGAGCAGCAGGAGAAGTGGTTGGGGAGAGCTAGAAGGAAGGAGATTATTGGATGTTATGCACAGACCGAGATGGGTCATGGTACCAATCTCCGAAAACTTGAAACCACAGCCACCTACTCCCCGGACACCCAGGAATTCATCCTGAACACCCCAACCATCACAGCTCTCAAATGGTGGCCCGGAGCACTCGGGAAGAGTTCCAACAACGCTATAGTAGTTGCTAATTTGCTTATCAAAGACCAAAACTATGGACCACATCCTTTCATGGTTCAACTGAGAGATGAGAAGACTCATATTCCACTAAAAGGAATCGTAGTTGGAGATATTGGCCCGAAAATGGCATTCAATGGAGCGGATAATGGATATCTTGGCTTCAATAACCACCGAATTCCAAGAACAAATCTTTTGATGAGACATACCAAAGTAGAAGCAAATGGGACTTATATAAAACCTTCTCATGCAAAGATTGGGTACTCGAGTATGGTAAAAGTGAGAAGTCGAATGGCAATGGATCAGGGATTGTTCTTAGCATCGGCTTTGGTAATAGCTGTGAGATACTCGGCAGTGAGGAGACAAGGTTTCTTGGAAGACAAAACCCAAAAAGTAAAAGTCCTTGACTACCAGACCCAGCAGCATCGTCTATTCCCATCATTAGCCCGTGCCTATGCATTTATATTTACTGGATTTGAGACAATTCACTTATATTCTCAATTATTAAAAGATGTTGATATGGGTAATACATCTGGAATGGCTGATTTACATGCATTGACTTCTGGTTTAAAATCTGTTGTTACTCATCAAACTGGAGAAGGTATCGAACAAGCGAGGATGGCTTGTGGTGAACATGGATATTCTATGGCTAGTTATATCTCTGAAATCTATGGAGTTGCAATTGGAGGATGTACTTATGAAGGGGAAAATATGGTAATGCTTCTGCAACTTGCACGGTATTTAGTGAAGTCCGTGGAACTTATCAAATCCGGAGAAGAAAAGAAGCTAGGACCAATGGTGTCATATCTAGCAGCTAAAGGTGGCCACCCTGATCTATCAAGTCTGAATGGATATGTAACAGCTTTCGAGCATATGGCTAGAAGGCAGGCTTGGAAAGCAACAGAGAAATTCTTGAAATTGATGGAAACTGGAGAATCAAGAGAAGTGGCGTGGAATAAATCAGCAGTGGAGTTGACTAGAGCTAGTCGTCTACACACCCGTCTATTCATAATCGAAGCTTTCATGAGAAGAGTGTCTCGAATTGAAGATATTCCAGTGAAAGAGGTTCTAACAGATCTTCTCCATCTTCATGTCAACTATGAACTATTGGATGTTGCCACGTATGCACTGGAATTCATGTCATCCACTCAGTTGGATTATATCAGAGATCAACTATATTTATATCTGGAGAAGATTCGACCAAGTGCAGTATCTCTAGTTGACTCTTTCCAGATTAGTGATATGCAATTGAGATCTGTTCTTGGCCGCCGGGATGGTAATGTCTACGAGAATCTCTTCAAGTGGGCAAAGTCGAGTCCCTTGAACAAATCTGATGTTCTACCGTCAGTCGATAAGTACTTGAAGCCTATGATGGAAAAGGCGAAGCTTTGAGGTACCCAATTCGCCCTATAGTGAGTCGTATTACGCGCGCTCACTGGCCGTCGTTTTACAACGTCGTGACTGGGAAAACCCTGGCGTTACCCAACTTAATCGCCTTGCAGCACATCCCCCTTTCGCCAGCTGGCGTAATAGCGAAGAGGCCCGCACCGATCGCCCTTCCCAACAGTTGCGCAGCCTGAATGGCGAATGGGACGCGCCCTGTAGCGGCGCATTAAGCGCGGCGGGTGTGGTGGTTACGCGCAGCGTGACCGCTACACTTGCCAGCGCCCTAGCGCCCGCTCCTTTCGCTTTCTTCCCTTCCTTTCTCGCCACGTTCGCCGGCTTTCCCCGTCAAGCTCTAAATCGGGGGCTCCCTTTAGGGTTCCGATTTAGTGCTTTACGGCACCTCGACCCCAAAAAACTTGATTAGGGTGATGGTTCACGTAGTGGGCCATCGCCCTGATAGACGGTTTTTCGCCCTTTGACGTTGGAGTCCACGTTCTTTAATAGTGGACTCTTGTTCCAAACTGGAACAACACTCAACCCTATCTCGGTCTATTCTTTTGATTTATAAGGGATTTTGCCGATTTCGGCCTATTGGTTAAAAAATGAGCTGATTTAACAAAAATTTAACGCGAATTTTAACAAAATATTAACGCTTACAATTTAGGTGGCACTTTTCGGGGAAATGTGCGCGGAACCCCTATTTGTTTATTTTTCTAAATACATTCAAATATGTATCCGCTCATGAGACAATAACCCTGATAAATGCTTCAATAATATTGAAAAAGGAAGAGTATGAGTATTCAACATTTCCGTGTCGCCCTTATTCCCTTTTTTGCGGCATTTTGCCTTCCTGTTTTTGCTCACCCAGAAACGCTGGTGAAAGTAAAAGATGCTGAAGATCAGTTGGGTGCACGAGTGGGTTACATCGAACTGGATCTCAACAGCGGTAAGATCCTTGAGAGTTTTCGCCCCGAAGAACGTTTTCCAATGATGAGCACTTTTAAAGTTCTGCTATGTGGCGCGGTATTATCCCGTATTGACGCCGGGCAAGAGCAACTCGGTCGCCGCATACACTATTCTCAGAATGACTTGGTTGAGTACTCACCAGTCACAGAAAAGCATCTTACGGATGGCATGACAGTAAGAGAATTATGCAGTGCTGCCATAACCATGAGTGATAACACTGCGGCCAACTTACTTCTGACAACGATCGGAGGACCGAAGGAGCTAACCGCTTTTTTGCACAACATGGGGGATCATGTAACTCGCCTTGATCGTTGGGAACCGGAGCTGAATGAAGCCATACCAAACGACGAGCGTGACACCACGATGCCTGTAGCAATGGCAACAACGTTGCGCAAACTATTAACTGGCGAACTACTTACTCTAGCTTCCCGGCAACAATTAATAGACTGGATGGAGGCGGATAAAGTTGCAGGACCACTTCTGCGCTCGGCCCTTCCGGCTGGCTGGTTTATTGCTGATAAATCTGGAGCCGGTGAGCGTGGGTCTCGCGGTATCATTGCAGCACTGGGGCCAGATGGTAAGCCCTCCCGTATCGTAGTTATCTACACGACGGGGAGTCAGGCAACTATGGATGAACGAAATAGACAGATCGCTGAGATAGGTGCCTCACTGATTAAGCATTGGTAACTGTCAGACCAAGTTTACTCATATATACTTTAGATTGATTTAAAACTTCATTTTTAATTTAAAAGGATCTAGGTGAAGATCCTTTTTGATAATCTCATGACCAAAATCCCTTAACGTGAGTTTTCGTTCCACTGAGCGTCAGACCCCGTAGAAAAGATCAAAGGATCTTCTTGAGATCCTTTTTTTCTGCGCGTAATCTGCTGCTTGCAAACAAAAAAACCACCGCTACCAGCGGTGGTTTGTTTGCCGGATCAAGAGCTACCAACTCTTTTTCCGAAGGTAACTGGCTTCAGCAGAGCGCAGATACCAAATACTGTCCTTCTAGTGTAGCCGTAGTTAGGCCACCACTTCAAGAACTCTGTAGCACCGCCTACATACCTCGCTCTGCTAATCCTGTTACCAGTGGCTGCTGCCAGTGGCGATAAGTCGTGTCTTACCGGGTTGGACTCAAGACGATAGTTACCGGATAAGGCGCAGCGGTCGGGCTGAACGGGGGGTTCGTGCACACAGCCCAGCTTGGAGCGAACGACCTACACCGAACTGAGATACCTACAGCGTGAGCTATGAGAAAGCGCCACGCTTCCCGAAGGGAGAAAGGCGGACAGGTATCCGGTAAGCGGCAGGGTCGGAACAGGAGAGCGCACGAGGGAGCTTCCAGGGGGAAACGCCTGGTATCTTTATAGTCCTGTCGGGTTTCGCCACCTCTGACTTGAGCGTCGATTTTTGTGATGCTCGTCAGGGGGGCGGAGCCTATGGAAAAACGCCAGCAACGCGGCCTTTTTACGGTTCCTGGCCTTTTGCTGGCCTTTTGCTCACATGTTCTTTCCTGCGTTATCCCCTGATTCTGTGGATAACCGTATTACCGCCTTTGAGTGAGCTGATACCGCTCGCCGCAGCCGAACGACCGAGCGCAGCGAGTCAGTGAGCGAGGAAGC

**Sequence S4. L4440-*acox-1.4* cDNA**

AACCTGGCTTATCGAAATTAATACGACTCACTATAGGGAGACCGGCAGATCTGATATCATCGATGAATTCGAGCTCCACCGCGGTGGCGGCCGCTCTAGAATGCATTTAAACACATCAATTTGCGAAGTTGATAACCCGGATCTCACCGAGGAACGGGAGAAGGGAACTTTTGATACGGATAAGATGGCGGCTGTGATCTATGGAAGTGAGAAGCTCGCCCGCCGGCGTCGAGAAATCTCAGAAGCTGTCTCAAAAATTCCGGAGCTAGCGGATACCCAACCCTTTCCGTTCATGGACCGACTTGAAAAAATTACAGAAGGATCCCGTAAACTAGAGGTGCTCAATAACAACATCCGTGATATAATTGACTACGATGATAACGGCGAGAGACTTCATATCTATCAAGAGGTTACGGGGATGGAAGGACACCCATTGGCTTTGCATGAGGTCATGTTTATTCCTGCATTAGTGTCACAGGCTAGCAAAGAGCAGCAGGAAAAATGGTTGGGGAGAGCTAGAAGGAGGGAGATTATTGGATGTTATGCACAGACCGAAATGGGTCACGGTACCAACCTCCGGAAACTGGAAACCACTGCCACCTACTTCCCAGACACCCAGGAATTTGTACTTAACACCCCAACGACTACAGCTCTCAAGTGGTGGCCCGGAGCTCTCGGGAAAAGTTCCAACTATGCAGTTGTGGTAGTTGATATGATTATCAAGGGAAAAAGCTATGGACCGCACCCCTTCATGGTTCAACTTCGAGATGAGAAGACTCATATTCCACTAAAAGGAATCGTAGTTGGAGATATTGGCCCGAAAATGTCATTTAATGGAGGTGACAATGGGTTCCTTGGATTCGATAAATTCCGAGTGCCCCGGACGAACCTTCTGATGCGGCATGTTAGGGTGGAAGCTGATGGAACATATGTTAAGCCTCCACATGCAAAGGTGAATCACTCTGCAATGGTTCATGTGAGAAGTCATATGGCAACAGGACAAGGGGCACTACTGGCACAGGCTTTGATTATTGCAGTGAGATATTCGGCAGTAAGAAGACAAGGGTTTTTGGAGAATAAAACCCAAGAAGTAAAAGTCCTTGACTACCAGACCCAGCAGCATCGTCTATTCCCATCATTAGCCCGTGCCTATGCATTTATATTTACTGGATTTGAGACAATTCACTTATATTCTCAATTATTAAAAGATGTTGATATGGGTAATACATCTGGAATGGCTGATTTACATGCATTGACTTCTGGTTTAAAATCTGTTGTTACTCATCAAACTGGAGAAGGTATTGAACAGGCGAGGATGGCTTGTGGTGGACATGGATACTCTATGGCTAGTTATATCTCTGAAATCTATGGAATTGCAATTGGAGGATGCACATACGAAGGAGAAAATATGGTGATGCTCCTGCAACTTGCACGGTATCTTGTCAAATCTGTAGAACTTATCAAATCCGGAGAAGCAAAGAAGCTAGGACCAATGGTGTCATATCTAGCAGCTAAAGGTGGCCACCCTGATCTATCAAGTCTGAATGGATATGTAACAGCTTTCGAGCATATGGCTAGAAGGCAAGCTTGGAAGGCAACTGAGAAATTCTTGAAATTGATGGAAAGTGGGGAATCGAGAGAAATAGCATGGAACAAATCAACAGTGGAGTTGACTAGAGCCAGTCGTCTACACACCCGTCTATTCATAATCGAAGCTTTCATGAGAAGAGTGTCTCGAATTGAAGATATTCCAGTGAAAGAGGTTCTAACAGATCTTCTCCATCTTCATGTCAACTATGAACTATTGGATGTTGCCACGTATGCACTGGAATTCATGTCATCCACTCAGTTGGATTATATCAGAGATCAACTATATTTATATCTGGAGAAGATTCGACCAAGTGCAGTATCTCTAGTTGACTCTTTCCAGATTAGTGATATGCAATTGAGATCTGTTCTTGGCCGCCGGGATGGTAATGTCTACGAGAATCTCTTCAAGTGGGCAAAGTCGAGTCCCTTGAACAAATCTGATGTTCTACCGTCAGTCGATAAGTACTTGATGCCTATGATGGAAAAGGCGAAACTTTAAGGTACCCAATTCGCCCTATAGTGAGTCGTATTACGCGCGCTCACTGGCCGTCGTTTTACAACGTCGTGACTGGGAAAACCCTGGCGTTACCCAACTTAATCGCCTTGCAGCACATCCCCCTTTCGCCAGCTGGCGTAATAGCGAAGAGGCCCGCACCGATCGCCCTTCCCAACAGTTGCGCAGCCTGAATGGCGAATGGGACGCGCCCTGTAGCGGCGCATTAAGCGCGGCGGGTGTGGTGGTTACGCGCAGCGTGACCGCTACACTTGCCAGCGCCCTAGCGCCCGCTCCTTTCGCTTTCTTCCCTTCCTTTCTCGCCACGTTCGCCGGCTTTCCCCGTCAAGCTCTAAATCGGGGGCTCCCTTTAGGGTTCCGATTTAGTGCTTTACGGCACCTCGACCCCAAAAAACTTGATTAGGGTGATGGTTCACGTAGTGGGCCATCGCCCTGATAGACGGTTTTTCGCCCTTTGACGTTGGAGTCCACGTTCTTTAATAGTGGACTCTTGTTCCAAACTGGAACAACACTCAACCCTATCTCGGTCTATTCTTTTGATTTATAAGGGATTTTGCCGATTTCGGCCTATTGGTTAAAAAATGAGCTGATTTAACAAAAATTTAACGCGAATTTTAACAAAATATTAACGCTTACAATTTAGGTGGCACTTTTCGGGGAAATGTGCGCGGAACCCCTATTTGTTTATTTTTCTAAATACATTCAAATATGTATCCGCTCATGAGACAATAACCCTGATAAATGCTTCAATAATATTGAAAAAGGAAGAGTATGAGTATTCAACATTTCCGTGTCGCCCTTATTCCCTTTTTTGCGGCATTTTGCCTTCCTGTTTTTGCTCACCCAGAAACGCTGGTGAAAGTAAAAGATGCTGAAGATCAGTTGGGTGCACGAGTGGGTTACATCGAACTGGATCTCAACAGCGGTAAGATCCTTGAGAGTTTTCGCCCCGAAGAACGTTTTCCAATGATGAGCACTTTTAAAGTTCTGCTATGTGGCGCGGTATTATCCCGTATTGACGCCGGGCAAGAGCAACTCGGTCGCCGCATACACTATTCTCAGAATGACTTGGTTGAGTACTCACCAGTCACAGAAAAGCATCTTACGGATGGCATGACAGTAAGAGAATTATGCAGTGCTGCCATAACCATGAGTGATAACACTGCGGCCAACTTACTTCTGACAACGATCGGAGGACCGAAGGAGCTAACCGCTTTTTTGCACAACATGGGGGATCATGTAACTCGCCTTGATCGTTGGGAACCGGAGCTGAATGAAGCCATACCAAACGACGAGCGTGACACCACGATGCCTGTAGCAATGGCAACAACGTTGCGCAAACTATTAACTGGCGAACTACTTACTCTAGCTTCCCGGCAACAATTAATAGACTGGATGGAGGCGGATAAAGTTGCAGGACCACTTCTGCGCTCGGCCCTTCCGGCTGGCTGGTTTATTGCTGATAAATCTGGAGCCGGTGAGCGTGGGTCTCGCGGTATCATTGCAGCACTGGGGCCAGATGGTAAGCCCTCCCGTATCGTAGTTATCTACACGACGGGGAGTCAGGCAACTATGGATGAACGAAATAGACAGATCGCTGAGATAGGTGCCTCACTGATTAAGCATTGGTAACTGTCAGACCAAGTTTACTCATATATACTTTAGATTGATTTAAAACTTCATTTTTAATTTAAAAGGATCTAGGTGAAGATCCTTTTTGATAATCTCATGACCAAAATCCCTTAACGTGAGTTTTCGTTCCACTGAGCGTCAGACCCCGTAGAAAAGATCAAAGGATCTTCTTGAGATCCTTTTTTTCTGCGCGTAATCTGCTGCTTGCAAACAAAAAAACCACCGCTACCAGCGGTGGTTTGTTTGCCGGATCAAGAGCTACCAACTCTTTTTCCGAAGGTAACTGGCTTCAGCAGAGCGCAGATACCAAATACTGTCCTTCTAGTGTAGCCGTAGTTAGGCCACCACTTCAAGAACTCTGTAGCACCGCCTACATACCTCGCTCTGCTAATCCTGTTACCAGTGGCTGCTGCCAGTGGCGATAAGTCGTGTCTTACCGGGTTGGACTCAAGACGATAGTTACCGGATAAGGCGCAGCGGTCGGGCTGAACGGGGGGTTCGTGCACACAGCCCAGCTTGGAGCGAACGACCTACACCGAACTGAGATACCTACAGCGTGAGCTATGAGAAAGCGCCACGCTTCCCGAAGGGAGAAAGGCGGACAGGTATCCGGTAAGCGGCAGGGTCGGAACAGGAGAGCGCACGAGGGAGCTTCCAGGGGGAAACGCCTGGTATCTTTATAGTCCTGTCGGGTTTCGCCACCTCTGACTTGAGCGTCGATTTTTGTGATGCTCGTCAGGGGGGCGGAGCCTATGGAAAAACGCCAGCAACGCGGCCTTTTTACGGTTCCTGGCCTTTTGCTGGCCTTTTGCTCACATGTTCTTTCCTGCGTTATCCCCTGATTCTGTGGATAACCGTATTACCGCCTTTGAGTGAGCTGATACCGCTCGCCGCAGCCGAACGACCGAGCGCAGCGAGTCAGTGAGCGAGGAAGC

**Sequence S5. L4440-*acox-1.5* cDNA**

AACCTGGCTTATCGAAATTAATACGACTCACTATAGGGAGACCGGCAGATCTGATATCATCGATGAATTCGAGCTCCACCGCGGTGGCGGCCGCTCTAGAATGCCGCTGAATAAGTTGATTCAAGATGGAGACAATCAAGATTTGACTGATGAAAGATTTAAAGCAACATTCGATACTGATGCTCTTGCCGCCGTGTTCCATGGAGGAGAAGATGCACTGAAAAGAATTCGAGAATTGAGAGATGAAGTAACCAAGCGGTGGCATTTATTTGATGCTCTTCCAGGTGCTCATAGGACAAGAGCCGAGAGAATGGAGGATGTCAGCAGGAAGCTGAAAAACCTCATGGAATCTGTGGGCGAGTTTGCTGATTTCACCAATAATCTCGATATGTTGGTTATCATTCGAGATGTAATGGGCATTGAAGGATTTCCATTGGCTCTTCATAACTTGATGTTTGTGCCAACTATTCAAAATCAAGCCGACGATGAGCAAACTGAGTGGTGGCTGATGGATGCACTTCAGGGCAAGATCATTGGAACTTATGCCCAAACGGAACTCGGACACGGAACCAATCTCGGAGCCATCGAAACTACAGCCACTTACGATAAGCTAACGGAAGAATTTATCATTCATACGCCAACTACAACGGCCACAAAATGGTGGCCTGGAGGTCTTGGAACCAGTTGTACACATGTCGTCCTTGTTGCAAACTTGATTATTGACACCAAAAACTATGGGCTTCATCCATTCTTCGTCCCAATTCGTGACCGTAATTCCTATTCTGTAATGTCTGGAGTACGTGTTGGAGATATTGGAACTAAAATGGGAGTGAACTGTGTCGATAACGGATTTTTGGCCTTTGACAACTACCGTATTCCAAGACGCAATATGCTTATGAAGCACAGTAAAGTATCAAAAGAAGGGCTCTACACGGCTCCATCTCATCCAAAAGTCGGATATACTACAATGTTATATATGAGATCTGAAATGATTTATCATCAAGCATATTATCTTGCAATGGCAATGGCTATTTCAATTCGATATTCAGCTGTTCGTAGACAAGGAGAGATTAAGCCAGGAACACAAGAAGTTCAAATTCTCGATTATCAAACTCAACAGTATCGTATTTTCCCGGGACTTGCTCGTTGTTTTGCTTTTAACACGGCAGCTGCAACTGTTCGTCAAATGACTGAAAACTGTATTAAACAACTTTCACATGGAAACTCTGATGTTCTTGCTGATTTGCATGCTCTTTCCTGTGGACTCAAGGCAGTTGTAACTCATCAAGCATCTCAATCAATTGATCAAGCAAGACAAGCATGTGGTGGACATGGATATTCGGATGCCAGTTATTTACCGACTTTGTATACTTGCTCGGTTGGTGCATGCACATATGAAGGAGAAAACATGGTAATGCTACTGCAGCTGTCCAAGTACCTAATGAAAGCGGCGGCAAAAGCCGAAAAGGGAGAAGAAATGGCACCATTGGTTGCATATCTTGTAAAACCGGACATTACCGAGACTAATGATAAGTTCGCAAAGATGCTCAGCCACTTCGAGCACATTGCACGTCATCGTGTAATGCATGCTTATCGTCAAATGATTGAAGAAGAGAAACAGGGAATCGAGAGAGATTATGCTTTTGCAAATCATTCAGTTGACTGGACTAAGGCTGCTCGTGCTCATACAAAGTTGTTTATCGCCCGTGGTTTCGTGAAAAGTGTGCAGGAAGTTAGTGATGAAGCAGTTCATGATGTACTTACAACACTTGCTGAACTCTATCTCTCTTATGAACTTATTGAAATGTCGGCTGATTTGACAGCTAATGGATATCTTTCCGAATCGGATGTCCAACAAATTCGACATCAAATCTACGATTCAATGAGAAAAACTCGCCGCAATGCTGTTTCAATTGTCGACTCATTTGATATTTGTGATCGAGAGCTTCGTTCAGTACTCGGTCGCCGTGATGGACATGTATATGAGAATCTGTACAAATGGGCTCAAATGAGCCCACTCAATGAGAGAAATCTTCCTCATGTTGAAAAGTATTTGAAGCCAATGACAAGCAAACTTTAAGGTACCCAATTCGCCCTATAGTGAGTCGTATTACGCGCGCTCACTGGCCGTCGTTTTACAACGTCGTGACTGGGAAAACCCTGGCGTTACCCAACTTAATCGCCTTGCAGCACATCCCCCTTTCGCCAGCTGGCGTAATAGCGAAGAGGCCCGCACCGATCGCCCTTCCCAACAGTTGCGCAGCCTGAATGGCGAATGGGACGCGCCCTGTAGCGGCGCATTAAGCGCGGCGGGTGTGGTGGTTACGCGCAGCGTGACCGCTACACTTGCCAGCGCCCTAGCGCCCGCTCCTTTCGCTTTCTTCCCTTCCTTTCTCGCCACGTTCGCCGGCTTTCCCCGTCAAGCTCTAAATCGGGGGCTCCCTTTAGGGTTCCGATTTAGTGCTTTACGGCACCTCGACCCCAAAAAACTTGATTAGGGTGATGGTTCACGTAGTGGGCCATCGCCCTGATAGACGGTTTTTCGCCCTTTGACGTTGGAGTCCACGTTCTTTAATAGTGGACTCTTGTTCCAAACTGGAACAACACTCAACCCTATCTCGGTCTATTCTTTTGATTTATAAGGGATTTTGCCGATTTCGGCCTATTGGTTAAAAAATGAGCTGATTTAACAAAAATTTAACGCGAATTTTAACAAAATATTAACGCTTACAATTTAGGTGGCACTTTTCGGGGAAATGTGCGCGGAACCCCTATTTGTTTATTTTTCTAAATACATTCAAATATGTATCCGCTCATGAGACAATAACCCTGATAAATGCTTCAATAATATTGAAAAAGGAAGAGTATGAGTATTCAACATTTCCGTGTCGCCCTTATTCCCTTTTTTGCGGCATTTTGCCTTCCTGTTTTTGCTCACCCAGAAACGCTGGTGAAAGTAAAAGATGCTGAAGATCAGTTGGGTGCACGAGTGGGTTACATCGAACTGGATCTCAACAGCGGTAAGATCCTTGAGAGTTTTCGCCCCGAAGAACGTTTTCCAATGATGAGCACTTTTAAAGTTCTGCTATGTGGCGCGGTATTATCCCGTATTGACGCCGGGCAAGAGCAACTCGGTCGCCGCATACACTATTCTCAGAATGACTTGGTTGAGTACTCACCAGTCACAGAAAAGCATCTTACGGATGGCATGACAGTAAGAGAATTATGCAGTGCTGCCATAACCATGAGTGATAACACTGCGGCCAACTTACTTCTGACAACGATCGGAGGACCGAAGGAGCTAACCGCTTTTTTGCACAACATGGGGGATCATGTAACTCGCCTTGATCGTTGGGAACCGGAGCTGAATGAAGCCATACCAAACGACGAGCGTGACACCACGATGCCTGTAGCAATGGCAACAACGTTGCGCAAACTATTAACTGGCGAACTACTTACTCTAGCTTCCCGGCAACAATTAATAGACTGGATGGAGGCGGATAAAGTTGCAGGACCACTTCTGCGCTCGGCCCTTCCGGCTGGCTGGTTTATTGCTGATAAATCTGGAGCCGGTGAGCGTGGGTCTCGCGGTATCATTGCAGCACTGGGGCCAGATGGTAAGCCCTCCCGTATCGTAGTTATCTACACGACGGGGAGTCAGGCAACTATGGATGAACGAAATAGACAGATCGCTGAGATAGGTGCCTCACTGATTAAGCATTGGTAACTGTCAGACCAAGTTTACTCATATATACTTTAGATTGATTTAAAACTTCATTTTTAATTTAAAAGGATCTAGGTGAAGATCCTTTTTGATAATCTCATGACCAAAATCCCTTAACGTGAGTTTTCGTTCCACTGAGCGTCAGACCCCGTAGAAAAGATCAAAGGATCTTCTTGAGATCCTTTTTTTCTGCGCGTAATCTGCTGCTTGCAAACAAAAAAACCACCGCTACCAGCGGTGGTTTGTTTGCCGGATCAAGAGCTACCAACTCTTTTTCCGAAGGTAACTGGCTTCAGCAGAGCGCAGATACCAAATACTGTCCTTCTAGTGTAGCCGTAGTTAGGCCACCACTTCAAGAACTCTGTAGCACCGCCTACATACCTCGCTCTGCTAATCCTGTTACCAGTGGCTGCTGCCAGTGGCGATAAGTCGTGTCTTACCGGGTTGGACTCAAGACGATAGTTACCGGATAAGGCGCAGCGGTCGGGCTGAACGGGGGGTTCGTGCACACAGCCCAGCTTGGAGCGAACGACCTACACCGAACTGAGATACCTACAGCGTGAGCTATGAGAAAGCGCCACGCTTCCCGAAGGGAGAAAGGCGGACAGGTATCCGGTAAGCGGCAGGGTCGGAACAGGAGAGCGCACGAGGGAGCTTCCAGGGGGAAACGCCTGGTATCTTTATAGTCCTGTCGGGTTTCGCCACCTCTGACTTGAGCGTCGATTTTTGTGATGCTCGTCAGGGGGGCGGAGCCTATGGAAAAACGCCAGCAACGCGGCCTTTTTACGGTTCCTGGCCTTTTGCTGGCCTTTTGCTCACATGTTCTTTCCTGCGTTATCCCCTGATTCTGTGGATAACCGTATTACCGCCTTTGAGTGAGCTGATACCGCTCGCCGCAGCCGAACGACCGAGCGCAGCGAGTCAGTGAGCGAGGAAGC
